# Melatonin and Female Fertility: A Systematic Review and Meta-Analysis of Clinical Supplementation, In Vitro Experiments, and Observational Evidence

**DOI:** 10.3390/nu18203431

**Published:** 2026-10-19

**Authors:** Hanqi Ye, Yijia Hu, Han Wang, Yumo Shi, Bei Wang, Jiechen Yin, Xiang Hong

**Affiliations:** Department of Epidemiology and Health Statistics, School of Public Health, Southeast University, Nanjing 210009, China; 15205860614@163.com (H.Y.); dingdang020211@163.com (Y.H.); muggleworld@163.com (H.W.); 15209865824@163.com (Y.S.); wangbeilxb@seu.edu.cn (B.W.); jiechen_yin@seu.edu.cn (J.Y.)

**Keywords:** melatonin, female fertility, dietary supplement, assisted reproductive technology, polycystic ovary syndrome, oocytes, embryo development

## Abstract

**Background/Objectives:** Melatonin has been proposed as a potential strategy to improve female fertility, but existing evidence is dispersed across assisted reproduction, reproductive disorders, and in vitro studies. This study aimed to comprehensively evaluate the relationships of melatonin supplementation, in vitro melatonin treatment, and endogenous melatonin levels with female fertility. **Methods:** PubMed, Web of Science, and EMBASE were searched from inception through February 2026. Randomized and non-randomized intervention studies, observational studies, and in vitro studies involving human oocytes or embryos were included. Random-effects meta-analyses were performed, and certainty of evidence was assessed using GRADE. **Results:** Thirty-six publications were included. Among women undergoing ART, melatonin supplementation increased the clinical pregnancy rate (RR = 1.55, 95% CI: 1.23–1.94) and live birth (RR = 1.59, 95% CI: 1.01–2.51). Melatonin supplementation also improved selected ovarian and embryological outcomes. In vitro treatment increased fertilization (OR = 1.97, 95% CI: 1.40–2.76) and blastocyst formation (OR = 2.00, 95% CI: 1.33–3.03). Observational studies showed higher morning serum melatonin levels in women with PCOS (MD = 7.83 pg/mL, 95% CI: 3.56–12.11). The certainty of evidence ranged from very low to moderate. **Conclusions:** Melatonin supplementation may improve selected reproductive outcomes, including clinical pregnancy and live birth, although evidence for live birth remains limited. In vitro and observational findings further support a potential role of melatonin in female reproductive function, but its clinical value requires confirmation in high-quality studies.

## 1. Introduction

Female fertility is an important component of reproductive health [1]. Reproductive disorders such as polycystic ovary syndrome (PCOS), diminished ovarian reserve (DOR), and premature ovarian insufficiency (POI) can adversely affect female fertility [2,3,4,5]. When impaired fertility manifests as infertility, assisted reproductive technology (ART) provides an important treatment option [6]. However, the likelihood of achieving pregnancy and live birth remains influenced by multiple factors [7]. Various strategies are therefore being explored to restore fertility and improve the success of ART.

Melatonin is an endogenous hormone primarily secreted by the pineal gland during the night, serving as a physiological signal of darkness and contributing to the regulation of circadian rhythms and the sleep–wake cycle [8]. In addition to these chronobiological functions, melatonin has potent antioxidant properties [9]. Oxidative stress is among the key biological factors influencing female fertility. Excessive reactive oxygen species can disrupt redox homeostasis in oocytes and embryos, impair oocyte maturation, fertilization, and subsequent embryo development, and thereby reduce reproductive potential [10]. Melatonin can directly scavenge free radicals, enhance antioxidant defenses, and contribute to the maintenance of mitochondrial homeostasis [11,12]. These diverse physiological and cellular actions have prompted interest in melatonin supplementation as a potential strategy to support female reproductive health.

Evidence regarding melatonin and female fertility has emerged from assisted reproduction, reproductive disorders, and in vitro studies of human oocytes and embryos [13]. However, existing studies vary considerably in population characteristics, study designs, and outcomes. Previous systematic reviews and meta-analyses have mainly focused on assisted reproduction, specific reproductive disorders, or individual experimental settings, whereas evidence spanning different reproductive contexts has largely been summarized in narrative reviews, and systematic integration across different study designs and levels of evidence remains lacking [13,14,15].

Therefore, this systematic review and meta-analysis aimed to comprehensively evaluate the relationships of exogenous melatonin supplementation, in vitro melatonin treatment, and endogenous melatonin levels with female fertility, and to assess the potential role and clinical relevance of melatonin in female fertility.

## 2. Materials and Methods

### 2.1. Search Strategy

PubMed, Web of Science, and EMBASE were systematically searched from database inception to February 2026. Search terms related to melatonin and female fertility were used, including assisted reproductive technology (ART), in vitro fertilization (IVF), intracytoplasmic sperm injection (ICSI), intrauterine insemination (IUI), polycystic ovary syndrome (PCOS), premature ovarian insufficiency (POI), premature ovarian failure (POF), and diminished ovarian reserve (DOR). Detailed search strategies are provided in Table S1. Reference lists of included studies were also screened. This review was registered with PROSPERO (CRD420261484630).

### 2.2. Eligibility Criteria

#### 2.2.1. Inclusion Criteria

Eligible studies included randomized controlled trials, non-randomized intervention studies, prospective cohort studies, case–control studies, cross-sectional studies, and in vitro studies using human oocytes or embryos derived from ART cycles. Eligible populations included women undergoing ART, women with PCOS, DOR, POF, or POI, and human oocytes or embryos exposed to melatonin in vitro. Studies were required to evaluate melatonin as an intervention, exposure, or biomarker and report at least one fertility-related, embryological, ovarian, or PCOS-related metabolic/endocrine outcome. Studies involving co-interventions were included only when the effect of melatonin could be evaluated separately.

#### 2.2.2. Exclusion Criteria

Non-original studies, animal studies, mechanistic or cell-line studies, non-English publications, duplicate reports, studies without extractable data, and studies without a comparable control group were excluded.

### 2.3. Study Selection and Data Extraction

Study selection and data extraction were independently performed by two reviewers. Any disagreements were resolved through discussion or consultation with a third reviewer. After duplicate removal, titles and abstracts were screened, followed by full-text assessment. Extracted data included study characteristics, population, melatonin exposure or intervention, comparator, relevant outcomes, corresponding sample sizes and outcome data, and information required for quality assessment.

The study selection process was conducted and reported in accordance with the PRISMA 2020 statement and is presented in Figure 1.

### 2.4. Assessment of Study Quality and Evidence Certainty

Study quality was independently assessed by two reviewers using design-specific tools: RoB 2.0 for RCTs [16]; ROBINS-I for non-randomized intervention studies [17]; the Newcastle–Ottawa Scale for observational studies [18,19]; and the QUIN tool for in vitro studies [20].

The overall certainty of evidence for key outcomes was assessed using GRADE, considering risk of bias, inconsistency, indirectness, imprecision, and publication bias [21].

### 2.5. Statistical Analysis

Statistical analyses were performed using R version 4.5.1. Continuous outcomes were pooled using mean differences (MDs), whereas dichotomous outcomes were pooled using risk ratios (RRs) or odds ratios (ORs). All effect estimates were reported with 95% confidence intervals (CIs). When continuous data were reported as medians with first and third quartiles, means and standard deviations were estimated using the method of Wan et al. [22]. For studies reporting means and 95% CIs without standard deviations, standard deviations were derived from the confidence intervals using the corresponding t distribution. For clinical outcomes, denominators followed the definitions used in the original studies and included participants/women, treatment cycles, or embryo-transfer cycles, as applicable. For in vitro studies, oocytes or embryos were used as the unit of analysis. Because donor-level data required to account for within-woman correlation were unavailable, cluster-adjusted estimates could not be reconstructed; therefore, unit-of-analysis characteristics and study-level results were additionally summarized descriptively (Table S5). For studies with multiple melatonin doses or concentrations, the 3 or 4 mg/day group for clinical studies and the 10^4^ nmol/L group for in vitro studies were selected for the primary analysis to align with the exposure levels used in single-dose studies and improve comparability across studies.

Studies or outcomes were not quantitatively pooled when the available data were insufficient for effect estimation, outcome denominators could not be reliably determined, measurement units could not be reliably harmonized, outcome definitions were not sufficiently comparable, or fewer than two studies provided suitable data for quantitative synthesis. Such findings were retained in the narrative synthesis where appropriate.

Random-effects models were used because of anticipated clinical and methodological heterogeneity. Between-study variance was estimated using the restricted maximum likelihood (REML) method, and 95% CIs for pooled effects were calculated using the conventional Wald method. Between-study heterogeneity was assessed using Cochran’s Q test and the *I*^2^ statistic. Subgroup analyses were conducted for selected outcomes with notable heterogeneity according to study design or melatonin concentration. Funnel plots were examined for outcomes including at least seven studies and interpreted cautiously because most analyses included fewer than 10 studies. Leave-one-out sensitivity analyses were performed for key outcomes. Additional sensitivity analyses were performed by excluding studies judged to be at high risk of bias using RoB 2.0 or at serious or critical risk of bias using ROBINS-I, where sufficient studies remained for pooling.

## 3. Results

### 3.1. Study Selection and Characteristics

A total of 2542 records were identified through the initial search. After screening, 36 publications met the eligibility criteria and were included in the review. The study selection process is shown in Figure 1.

The included studies comprised randomized controlled trials, non-randomized intervention studies, observational studies, and in vitro studies using human oocytes or embryos. Study populations and experimental materials included women undergoing assisted reproductive technology, women with polycystic ovary syndrome, women with diminished ovarian reserve or premature ovarian failure, and human oocytes and embryos. Studies of exogenous melatonin mainly evaluated the effects of melatonin supplementation on assisted reproductive outcomes, ovarian function, and PCOS-related outcomes; in vitro studies assessed the effects of different melatonin concentrations on oocyte fertilization and embryo development; and observational studies investigated associations between endogenous melatonin levels and reproductive disorders. Detailed characteristics of the included studies are presented in Table S2, and the corresponding outcome-level extracted data are provided in Table S8.

### 3.2. Quality Assessment of Included Studies

The results of quality assessment varied across study designs. Risk-of-bias assessments of RCTs varied from low risk to high risk, with concerns primarily related to the randomization process, deviations from intended interventions, and selective reporting of results (Figure S1). Non-randomized intervention studies had a relatively higher risk of bias, mainly due to inadequate control of confounding and bias related to participant selection (Figure S2). Observational studies were generally of moderate quality, with the main limitations being insufficient comparability between groups and potential confounding (Table S3). In vitro studies generally showed a low or moderate risk of bias (Table S4).

### 3.3. Meta-Analysis Results

#### 3.3.1. Clinical Effects of Exogenous Melatonin Supplementation

##### ART Population

Among women undergoing ART, melatonin supplementation increased the clinical pregnancy rate (RR = 1.55, 95% CI: 1.23–1.94), biochemical pregnancy rate (RR = 1.61, 95% CI: 1.09–2.38) and live birth (RR = 1.59, 95% CI: 1.01–2.51). Melatonin supplementation also increased the number of mature oocytes (MD = 2.62, 95% CI: 0.36–4.88), and transferable embryos (MD = 4.51, 95% CI: 2.59–6.43), while no significant differences were found in the number of oocytes retrieved, follicular fluid estradiol concentration, fertilized oocytes, fertilization rate, good-quality embryos, or day 3 good-quality embryos (Table 1; Figure 2 and Figure S3). Miscarriage was not quantitatively pooled because the two studies reporting this outcome used different denominators. The two studies showed effects in opposite directions [23,24].

Subgroup analyses by study design showed a larger increase in oocytes retrieved in RCTs than in non-randomized intervention studies (*p* = 0.040), whereas no significant subgroup difference was observed for mature oocytes (*p* = 0.875) (Figure 3).

##### PCOS Population

Among women with PCOS, pooled analyses showed no significant effects of melatonin supplementation on total antioxidant capacity, fasting glucose, or lipid-related outcomes (Table 2; Figure S4).

In addition to these outcomes, Akter et al. reported improvements in follicular development, ovulation, and clinical pregnancy [33]; Jamilian et al. and Alizadeh et al. reported improvements in hyperandrogenism-related outcomes [34,35]; and Shabani et al. reported improvements in sleep and psychological outcomes [36].

##### DOR/POF Population

Among women with DOR, pooled analyses showed that melatonin supplementation increased the number of mature oocytes (MD = 2.70, 95% CI: 0.86–4.54) and serum estradiol levels on the day of hCG administration (MD = 694.55 pg/mL, 95% CI: 401.65–987.44), whereas no significant effects were observed on total HMG dose or stimulation duration (Table 3; Figure S5). In addition, Sadeghpour et al. reported improvements in oocyte retrieval, fertilization, embryo quality, and biochemical pregnancy [37], while Jahromi et al. reported improvements in mature oocyte retrieval and good-quality embryos [38]. Among women with POF, Li et al. reported reductions in LH, FSH, and ROS levels and improved ovarian hormone secretion following melatonin treatment [39].

#### 3.3.2. Effects of Melatonin on Oocytes and Embryos in In Vitro Studies

In vitro melatonin treatment increased fertilization, oocyte survival, embryo quality, and blastocyst-related outcomes, whereas no significant effects were observed on oocyte maturation or cleavage rates (Table 4; Figure 4 and Figure S6). One study not included in the quantitative meta-analysis reported that 1 nM melatonin increased nuclear maturation of GV- and MI-stage oocytes, whereas 10^6^ nM reduced maturation [40].

To explore the effects of different melatonin concentrations, subgroup analyses were further conducted (Figure 5). The results showed no significant differences in fertilization or blastocyst formation rates across melatonin concentrations.

Among the 10 in vitro studies, only seven reported donor/woman numbers for the experiments corresponding to the developmental outcomes included in this review, and only one explicitly reported sibling-oocyte allocation across experimental conditions for the main comparison. None reported statistical adjustment for within-woman clustering or pairing. Study-level effect estimates were therefore additionally presented descriptively alongside the unit-of-analysis characteristics in Table S5.

#### 3.3.3. Association Between Endogenous Melatonin Levels and Fertility

Among women with PCOS, morning serum melatonin levels were higher than in controls (MD = 7.83 pg/mL, 95% CI: 3.56–12.11; *I*^2^ = 22.7%), whereas no significant difference was observed in nighttime serum melatonin levels (Table 5; Figure S7). Abduljabbar et al., which was not included in the quantitative synthesis because its reported measurement unit could not be reliably harmonized, also reported higher morning serum melatonin levels in women with PCOS than in controls [49].

In addition to serum melatonin levels, Shreeve et al. and Luboshitzky et al. reported higher urinary melatonin metabolite levels in women with PCOS [50,51]. Li et al. found lower follicular fluid melatonin concentrations, which were positively associated with day 3 good-quality embryo formation [52].

### 3.4. Sensitivity Analyses and Publication Bias

Leave-one-out sensitivity analyses showed stable pooled estimates for clinical pregnancy (RR range: 1.50–1.65), fertilization (OR range: 1.79–2.19), and blastocyst formation (OR range: 1.84–2.27). The statistical significance of the number of oocytes retrieved and mature oocytes varied after exclusion of some studies (Table S6).

Additional sensitivity analyses excluding studies at high, serious, or critical risk of bias showed that the associations with clinical pregnancy (RR = 1.73, 95% CI: 1.27–2.36) remained statistically significant. For the number of oocytes retrieved, the pooled estimate became statistically significant (MD = 1.00, 95% CI: 0.23–1.76), while heterogeneity decreased to 0%. In contrast, the association with the number of mature oocytes was no longer statistically significant (MD = 3.65, 95% CI: −0.47–7.78), with substantial heterogeneity remaining (*I*^2^ = 97.6%). Estimates for the number of fertilized oocytes remained nonsignificant. After these exclusions, too few studies remained to quantitatively synthesize the number of transferable embryos, the number of good-quality embryos, and biochemical pregnancy rate (Table S6).

Funnel plots showed no obvious asymmetry for the key outcomes (Figure 6). However, given the limited number of included studies, publication bias cannot be completely ruled out.

### 3.5. Certainty of Evidence

The certainty of evidence ranged from very low to moderate (Table S7). Evidence for clinical pregnancy from RCTs was rated as moderate, whereas the evidence for live birth was rated as low. Evidence for the other key outcomes was generally low or very low, mainly because of risk of bias, limited sample size, and heterogeneity.

## 4. Discussion

### 4.1. Main Findings

This study integrated evidence from exogenous clinical interventions, in vitro studies of human oocytes and embryos, and observational studies of endogenous melatonin. Overall, exogenous melatonin supplementation showed potential benefits for selected assisted reproductive outcomes; in vitro studies further supported direct effects of melatonin on human oocyte and embryo development; and altered endogenous melatonin levels in women with PCOS suggested that melatonin homeostasis may be related to female fertility.

In studies of exogenous melatonin supplementation, meta-analysis showed higher clinical and biochemical pregnancy rates with low between-study heterogeneity. A higher live birth rate was also observed; however, this finding should be interpreted cautiously because the certainty of evidence was low and the confidence interval was wide, with its lower bound close to the null. Melatonin supplementation was also associated with increases in the number of mature oocytes and transferable embryos. However, the evidence for these ovarian response and intermediate embryological outcomes should be interpreted cautiously, given the substantial heterogeneity observed for some outcomes and the loss of statistical significance for mature oocyte number after studies at high, serious, or critical risk of bias were excluded. Among women with DOR, melatonin supplementation increased serum estradiol levels on the day of hCG administration. Estradiol is primarily secreted by follicular granulosa cells, and this increase may reflect enhanced follicular endocrine activity during ovarian stimulation, although whether this indicates improved follicular developmental potential requires further evidence [53,54]. In contrast, pooled metabolic and oxidative stress-related outcomes in women with PCOS showed no clear improvement, and few studies directly assessed fertility outcomes; therefore, the reproductive benefit of melatonin supplementation in women with PCOS cannot yet be determined.

In vitro studies provided further evidence for direct effects of melatonin on oocytes and embryos. Meta-analysis showed that melatonin treatment increased fertilization rate and improved outcomes related to good-quality embryos and blastocysts, while no significant effects were observed on oocyte maturation or cleavage rates. This pattern suggests that the potential effects of melatonin may be more closely related to fertilization competence and subsequent embryo developmental potential rather than a general enhancement of development at all stages. Notably, these effects may be concentration dependent, as one included study reported a reduced oocyte maturation rate at 10^6^ nM melatonin, indicating that higher concentrations do not necessarily confer greater developmental benefit. Previous studies have suggested that the potential effects of melatonin on oocyte and embryo development may involve multiple biological pathways. Melatonin may act through receptor-mediated signaling at the ovarian cell membrane as well as through receptor-independent pathways [55,56]. Melatonin can cross cell membranes and enter intracellular and subcellular compartments to scavenge reactive oxygen species and reduce oxidative damage, while also supporting cellular energy metabolism by maintaining mitochondrial membrane potential and ATP production [11,57]. These actions may help preserve redox and mitochondrial homeostasis in oocytes, thereby providing a more favorable intracellular environment for fertilization and early embryo development.

Melatonin is not only an exogenous intervention but also an endogenous component of female reproductive physiology [58,59]. In women with PCOS, morning serum melatonin levels were higher than in controls, whereas no significant difference was observed in nighttime serum melatonin levels, with substantial heterogeneity across studies. Nighttime urinary melatonin metabolite levels were also increased, while follicular fluid melatonin concentrations were reduced and positively associated with the day 3 good-quality embryo formation rate. Together, these findings may reflect altered temporal regulation of melatonin in PCOS rather than a uniform increase in melatonin levels; however, differences in sampling time, assay methods, and collection conditions limit a more definitive interpretation. The findings in follicular fluid further suggest that local melatonin status within the ovarian microenvironment may be related to oocyte and embryo developmental potential. However, studies directly comparing the reproductive relevance of circulating and local melatonin levels are currently lacking. Overall, the endogenous evidence indicates that melatonin may have physiological and pathological relevance to female reproduction beyond its potential value as an exogenous intervention.

### 4.2. Clinical Implications

The findings of this study suggest that melatonin may have potential relevance to female reproductive health, particularly in the context of assisted reproduction. Potential benefits were observed across both laboratory and clinical outcomes, including selected oocyte-related and embryo-related outcomes as well as biochemical pregnancy, clinical pregnancy, and live birth. However, given the limited number of studies and the overall certainty of the current evidence, routine melatonin supplementation for fertility treatment cannot yet be recommended.

An important unresolved issue is the relationship between oral melatonin dose and local exposure within the follicular microenvironment. Although clinical studies have shown that oral melatonin supplementation can increase follicular fluid melatonin concentrations [25,26], the relationship among oral dose, follicular exposure, and the concentrations associated with biological effects in vitro remains poorly defined. Establishing this dose–exposure–effect relationship may therefore be important for determining clinically appropriate dosing regimens.

The timing of supplementation may be equally important. Melatonin is not merely an antioxidant but also a physiological signal involved in the regulation of circadian rhythms and the sleep–wake cycle. Accordingly, the effects of supplementation may depend not only on the administered dose but also on its timing relative to the endogenous circadian rhythm. Inappropriate timing may alter normal circadian signaling and contribute to adverse effects such as drowsiness. Future clinical studies should therefore consider both dose and timing when optimizing melatonin supplementation.

Although PCOS has recently been renamed polyendocrine metabolic ovarian syndrome (PMOS), the studies included in this review enrolled participants according to the PCOS definitions and diagnostic criteria used at the time [60]. Therefore, we retained the term PCOS for these study populations rather than retrospectively reclassifying them as PMOS. Future studies should consider the updated PMOS framework when defining study populations and interpreting their comparability with previous PCOS-based evidence.

Given the heterogeneity of the populations studied, including women undergoing ART and those with DOR or PCOS, the effects of melatonin should not be assumed to be uniform across reproductive conditions. Altered endogenous melatonin status in disorders such as PCOS further suggests that reproductive phenotype, metabolic and endocrine characteristics, and endogenous melatonin status may all be relevant when identifying populations most likely to benefit from supplementation. Future studies should therefore explore more individualized supplementation strategies that incorporate these characteristics.

Overall, before melatonin can be recommended for routine use in assisted reproduction, higher-quality evidence is needed to establish the optimal dose and timing of administration and to identify the target populations most likely to benefit.

### 4.3. Strengths and Limitations

This study has several strengths. Compared with previous studies that mainly focused on a single population or outcome, we integrated evidence from clinical interventions, in vitro experiments, and observational studies, thereby evaluating the relationship between melatonin and female fertility across multiple levels, including clinical outcomes, oocyte and embryo development, and disease-related melatonin status. Design-specific quality assessment and GRADE were used to evaluate study quality and evidence certainty.

However, several limitations should be acknowledged. The small number of studies available for some outcomes limited the reliability of further subgroup analyses and publication bias assessment. Differences in study design, study populations, intervention regimens, and outcome definitions also increased heterogeneity and uncertainty in interpretation. For the in vitro studies, oocytes or embryos from the same woman may not have been statistically independent. Because donor-level data and the information required to account for within-woman correlation were unavailable, cluster-adjusted estimates could not be reconstructed. Consequently, the confidence intervals of the pooled in vitro estimates may be overly precise, and these results should be interpreted cautiously. For studies of endogenous melatonin, differences in sampling time, assay methods, collection conditions, and reported measurement units limited comparability across studies and, in some cases, precluded quantitative pooling. In addition, evidence from non-randomized, in vitro, and observational studies limited the overall certainty of evidence, and observational findings on endogenous melatonin do not permit causal inference. Sensitivity analyses further indicated that some findings were not fully robust to the exclusion of studies at high, serious, or critical risk of bias, particularly the number of mature oocytes.

Larger randomized trials with standardized protocols and core outcomes are needed to clarify the clinical value of melatonin supplementation. Greater standardization of experimental and measurement methods is also needed to improve the comparability of in vitro and endogenous melatonin studies.

## 5. Conclusions

The present study suggests that melatonin is associated with female fertility and that melatonin supplementation may improve selected reproductive outcomes, particularly clinical pregnancy in women undergoing assisted reproduction, with a potential benefit also observed for live birth, although evidence for this outcome remains limited. In vitro studies further support potential effects of melatonin on fertilization and early embryo development, while altered endogenous melatonin levels in women with PCOS suggest a possible role of melatonin homeostasis in female reproductive function. However, given the limited number of studies for some outcomes, the methodological limitations of the available evidence and its generally low certainty, current evidence is insufficient to support routine melatonin supplementation for fertility treatment, and its clinical value requires further evaluation in high-quality studies.

## Figures and Tables

**Figure 1:**
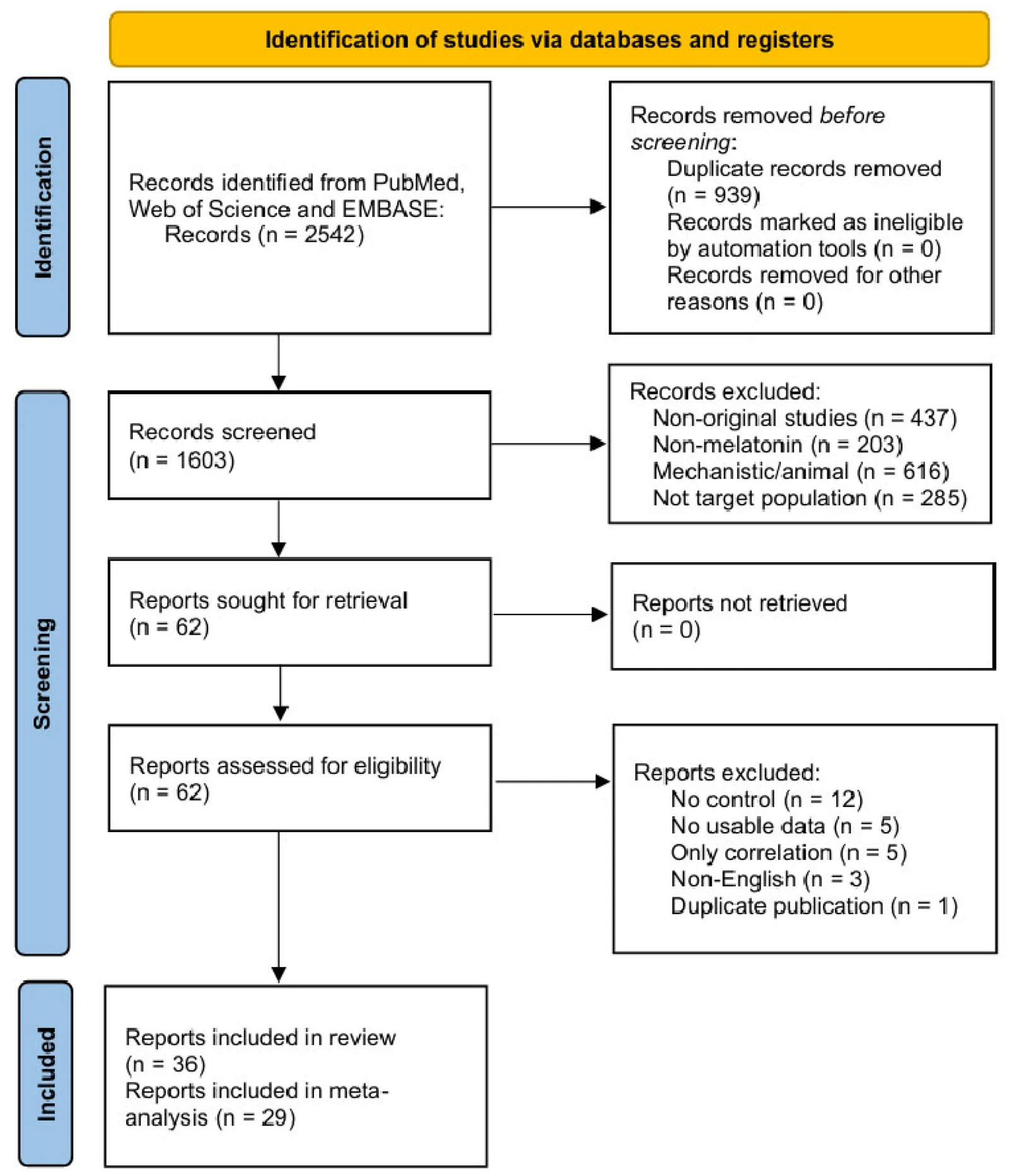
PRISMA flow diagram of the study selection process.

**Figure 2:**
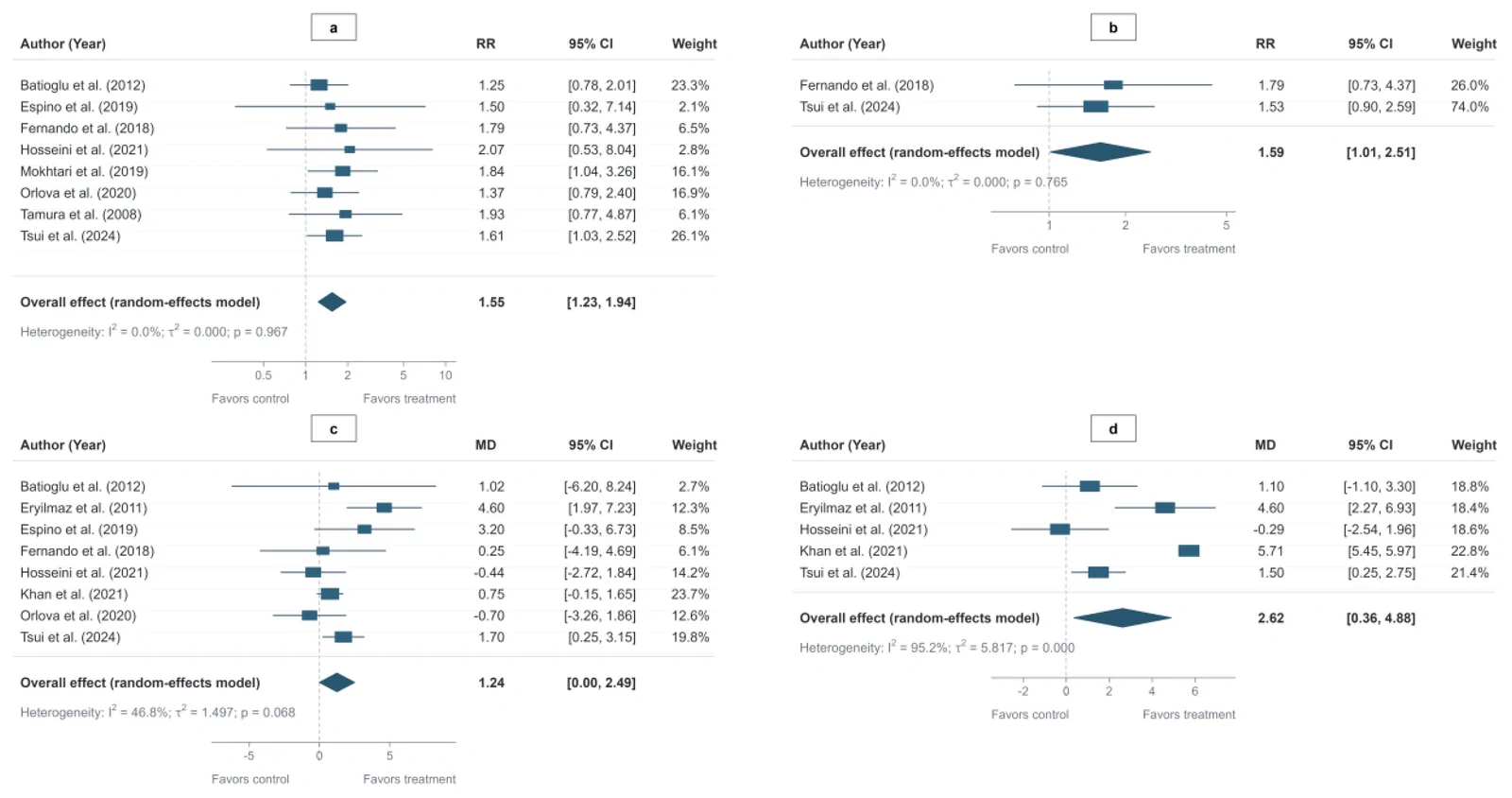
Forest plots of melatonin supplementation on ART outcomes: (**a**) clinical pregnancy rate; (**b**) live birth rate; (**c**) number of oocytes retrieved; (**d**) number of mature oocytes [23,24,25,26,27,28,29,30,31,32].

**Figure 3:**
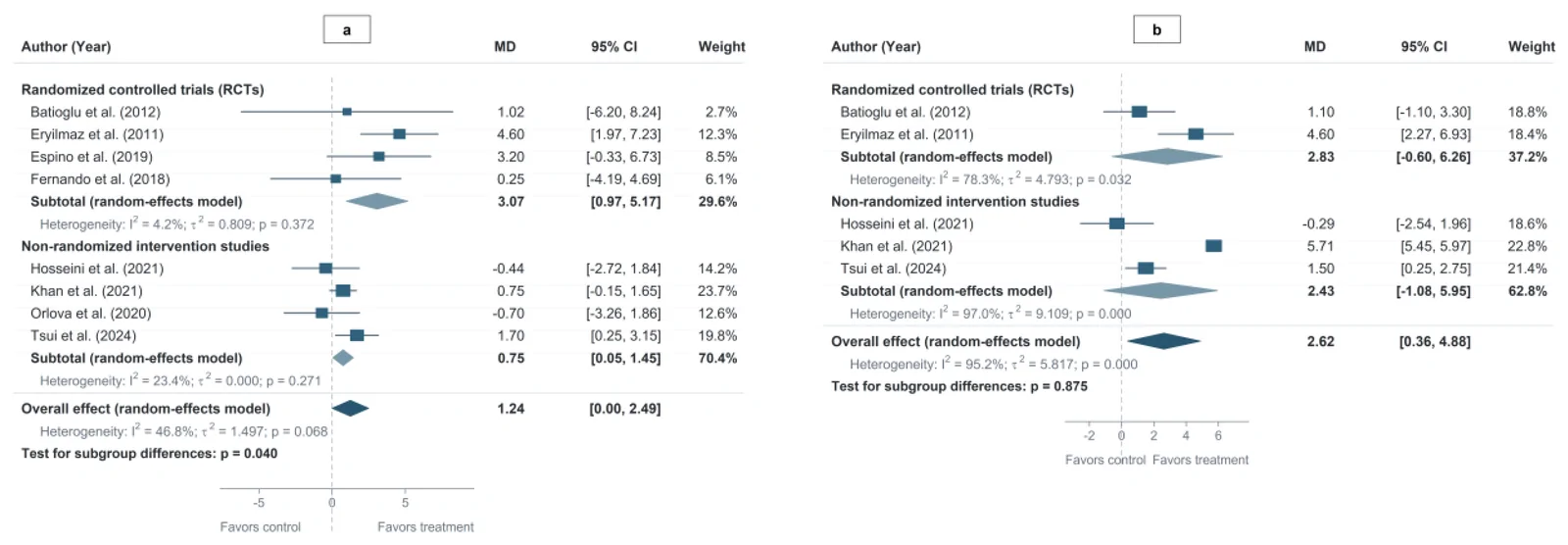
Subgroup analyses by study design: (**a**) number of oocytes retrieved; (**b**) number of mature oocytes [23,24,25,26,27,28,29,32].

**Figure 4:**
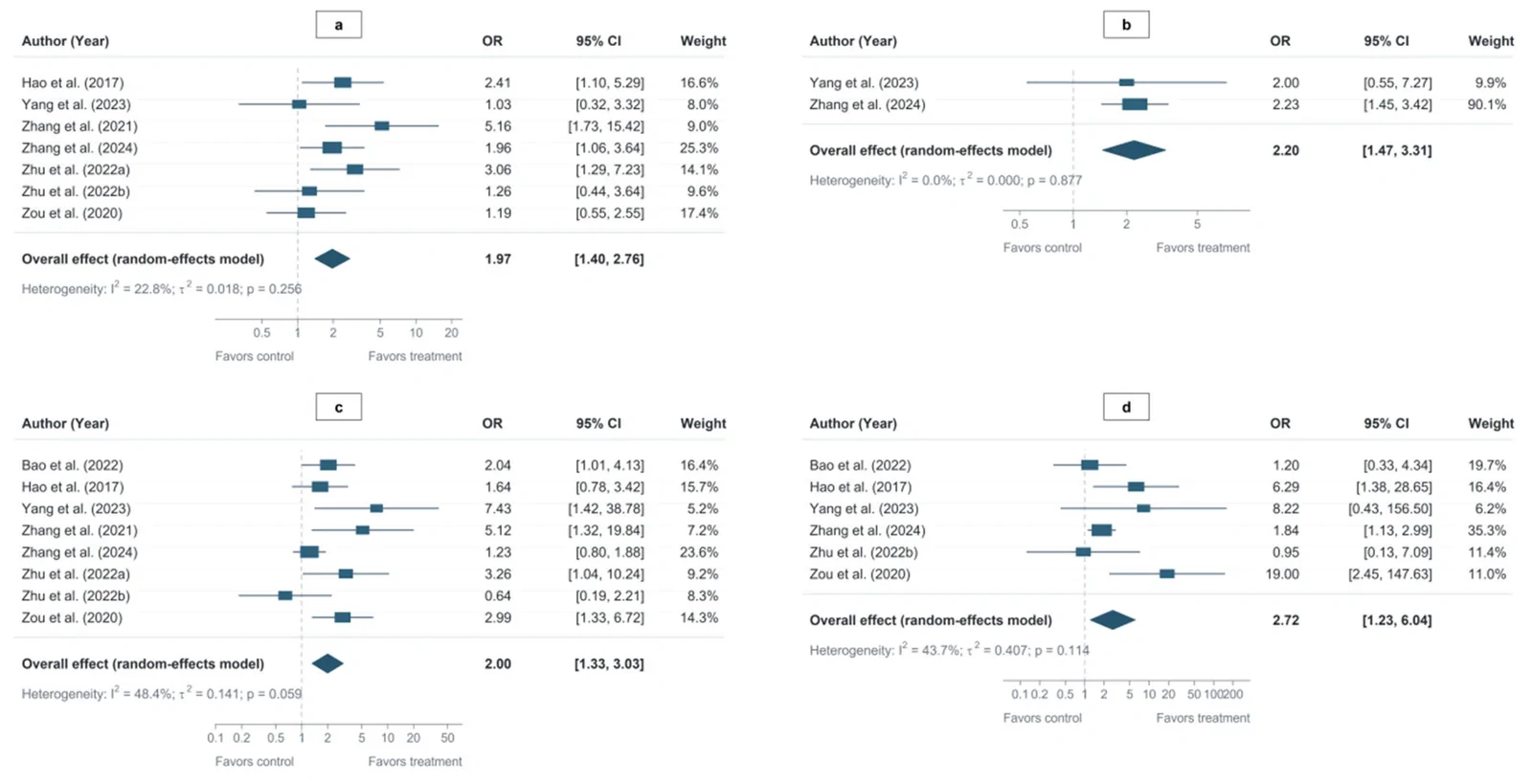
Forest plots of melatonin treatment on oocyte and embryo developmental outcomes: (**a**) fertilization rate; (**b**) good-quality embryo rate; (**c**) blastocyst formation rate; (**d**) good-quality blastocyst rate [41,42,43,44,45,46,47,48].

**Figure 5:**
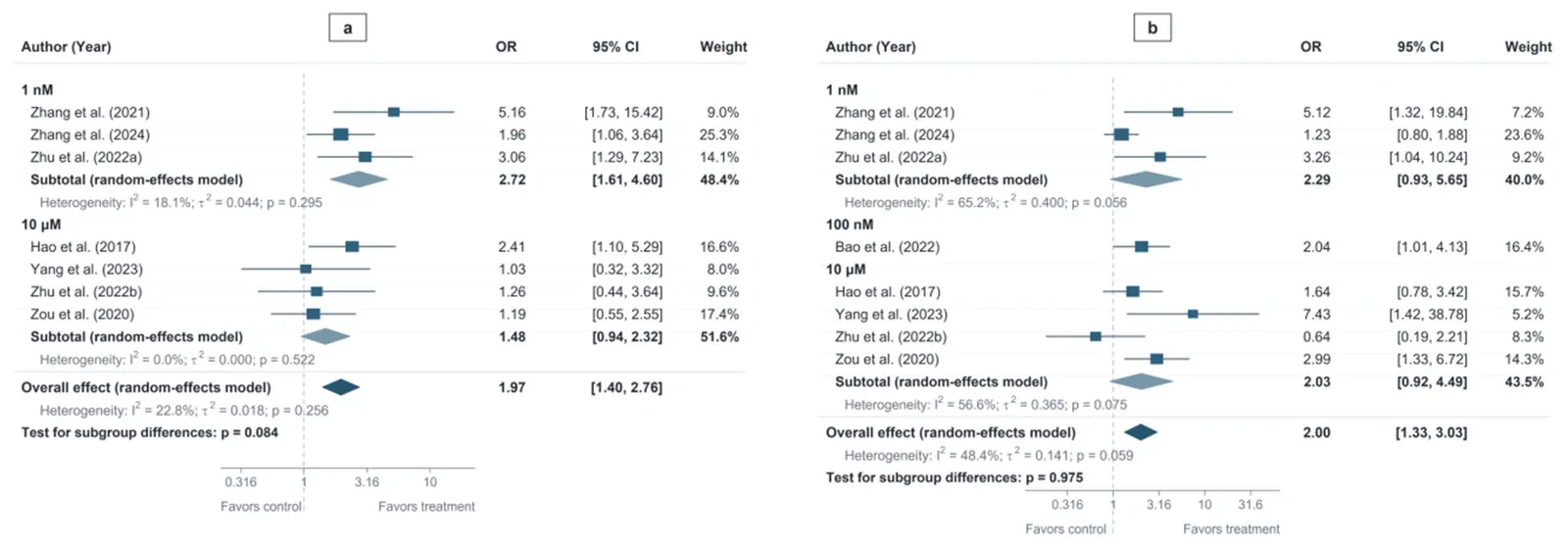
Subgroup analyses by melatonin concentration: (**a**) fertilization rate; (**b**) blastocyst formation rate [41,42,43,44,45,46,47,48].

**Figure 6:**
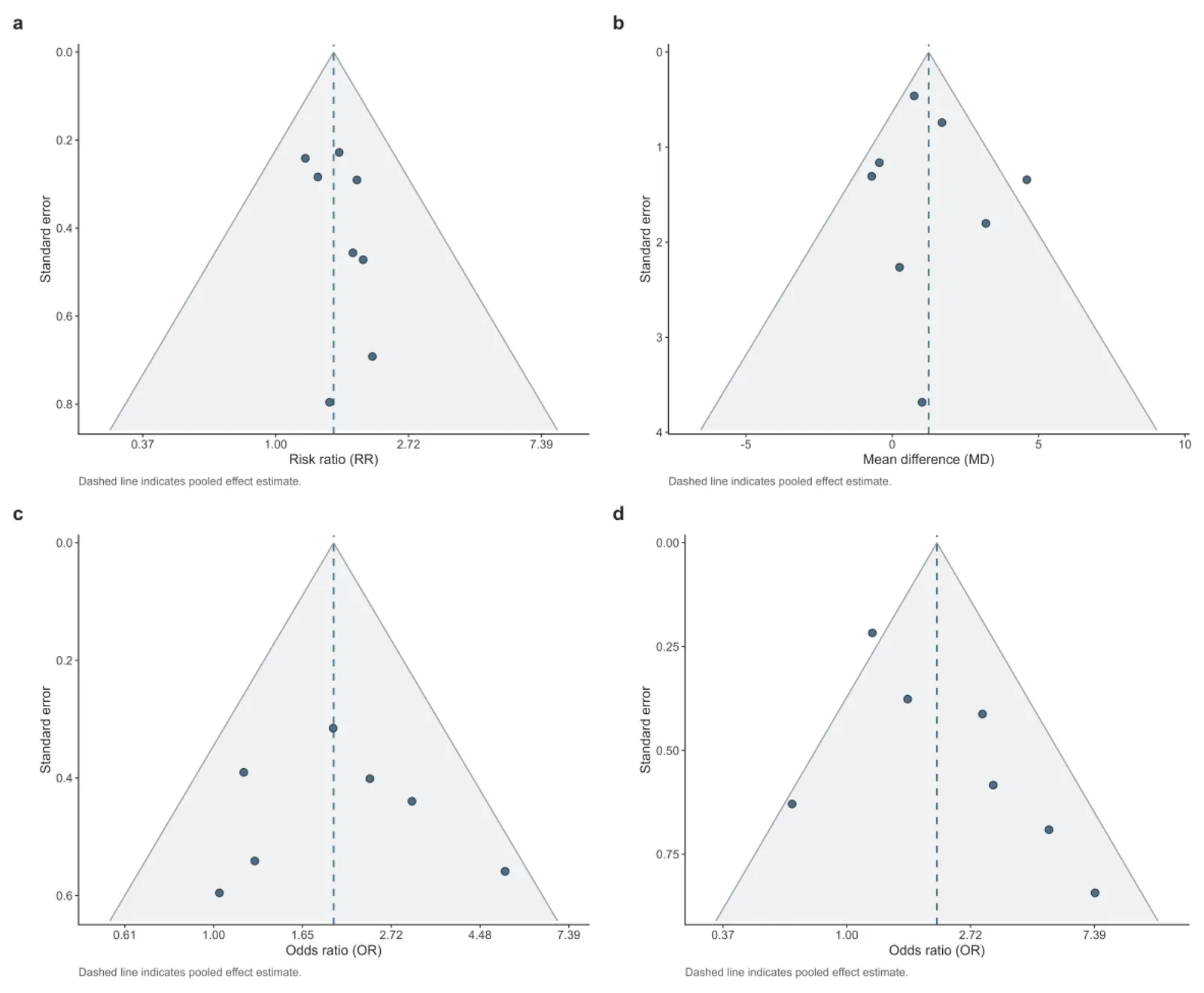
Funnel plots for assessment of potential publication bias in key outcomes: (**a**) clinical pregnancy rate; (**b**) number of oocytes retrieved; (**c**) fertilization rate; (**d**) blastocyst formation rate.

**Table 1:** Meta-analysis of melatonin supplementation on ART outcomes.

Outcome	No. of Studies	Pooled Effect Under Random-Effects Model (95% CI)	*p* Value	*I* ^2^
Number of oocytes retrieved	8	MD 1.24 (−0.004–2.49)	0.051	46.8%
Number of mature oocytes	5	MD 2.62 (0.36–4.88)	0.023	95.2%
Number of fertilized oocytes	3	MD 2.73 (−1.13–6.60)	0.165	93.1%
Fertilization rate (%)	2	MD 13.53 (−12.43–39.48)	0.307	94.1%
Number of transferable embryos	2	MD 4.51 (2.59–6.43)	<0.001	64.5%
Number of good-quality embryos	3	MD 0.45 (−0.45–1.35)	0.327	0.0%
Number of day 3 good-quality embryos	2	MD 2.94 (−1.63–7.50)	0.208	99.5%
Follicular fluid estradiol concentration	2	MD 79.95 (−134.40–294.30)	0.465	95.3%
Clinical pregnancy rate	8	RR 1.55 (1.23–1.94)	<0.001	0.0%
Biochemical pregnancy rate	4	RR 1.61 (1.09–2.38)	0.018	0.0%
Live birth rate	2	RR 1.59 (1.01–2.51)	0.046	0.0%

**Table 2:** Meta-analysis of melatonin supplementation on PCOS-related outcomes.

Outcome	No. of Studies	Pooled MD Under Random-Effects Model (95% CI)	*p* Value	*I* ^2^
Total antioxidant capacity	2	50.85 (−52.80–154.49)	0.336	95.7%
Fasting blood glucose (mg/dL)	2	−0.59 (−2.39–1.21)	0.522	0.0%
Triglycerides (mg/dL)	2	−2.54 (−20.61–15.52)	0.782	39.8%
Total cholesterol (mg/dL)	2	−1.56 (−16.01–12.90)	0.833	78.1%
Low-density lipoprotein cholesterol (mg/dL)	2	−1.98 (−11.57–7.61)	0.686	61.8%
High-density lipoprotein cholesterol (mg/dL)	2	0.49 (−1.42–2.40)	0.618	31.9%

**Table 3:** Meta-analysis of melatonin supplementation on DOR-related outcomes.

Outcome	No. of Studies	Pooled MD Under Random-Effects Model (95% CI)	*p* Value	*I* ^2^
Number of mature oocytes	2	2.70 (0.86–4.54)	0.004	86.4%
Serum estradiol concentration on the day of hCG administration (pg/mL)	2	694.55 (401.65–987.44)	<0.001	0%
Total HMG dose (IU)	2	112.67 (−236.29–461.62)	0.527	0%
Duration of ovarian stimulation	2	−0.54 (−2.87–1.79)	0.647	92.7%

**Table 4:** Meta-analysis of melatonin treatment on oocyte and embryo developmental outcomes.

Outcome	No. of Studies	Pooled OR Under Random-Effects Model (95% CI)	*p* Value	*I* ^2^
Oocyte survival rate	2	3.10 (1.53–6.29)	0.002	0%
Oocyte maturation rate	4	1.65 (0.82–3.29)	0.159	73.8%
Fertilization rate	7	1.97 (1.40–2.76)	<0.001	22.8%
Cleavage rate	7	1.24 (0.79–1.94)	0.349	0%
Day 3 good-quality embryo rate	2	4.02 (1.94–8.33)	<0.001	0%
Good-quality embryo rate	2	2.20 (1.47–3.31)	<0.001	0%
Blastocyst formation rate	8	2.00 (1.33–3.03)	<0.001	48.4%
Good-quality blastocyst rate	6	2.72 (1.23–6.04)	0.014	43.7%

**Table 5:** Meta-analysis of endogenous melatonin levels in women with polycystic ovary syndrome.

Outcome	No. of Studies	Pooled MD Under Random-Effects Model (95% CI)	*p* Value	*I* ^2^
Morning serum melatonin level (pg/mL)	2	7.83 (3.56–12.11)	<0.001	22.7%
Nighttime serum melatonin level (pg/mL)	2	8.05 (−36.49–52.59)	0.723	99.6%

## Data Availability

All data analyzed in this study were obtained from previously published studies and are available within the article and its Supplementary Materials.

## Supplementary Materials

The following supporting information can be downloaded at: https://www.mdpi.com/article/10.3390/nu18203431/s1, Figure S1: Risk of bias assessment of RCTs using RoB 2. (a) Risk-of-bias judgments for individual studies across domains; (b) summary of risk-of-bias judgments across domains; Figure S2: Risk of bias assessment of non-randomized studies using ROBINS-I. (a) Risk-of-bias judgments for individual studies across domains; (b) summary of risk-of-bias judgments across domains; Figure S3: Forest plots of melatonin supplementation on ART outcomes. (a) follicular fluid estradiol concentration; (b) fertilization rate; (c) number of fertilized oocytes; (d) number of good-quality embryos; (e) number of day 3 good-quality embryos; (f) number of transferable embryos; (g) biochemical pregnancy rate; Figure S4: Forest plots of melatonin supplementation on PCOS-related outcomes. (a) total antioxidant capacity; (b) fasting blood glucose; (c) triglycerides; (d) total cholesterol; (e) low-density lipoprotein cholesterol; (f) high-density lipoprotein cholesterol; Figure S5: Forest plots of melatonin supplementation on DOR-related outcomes. (a) duration of ovarian stimulation; (b) total hMG dose; (c) serum estradiol concentration on the day of hCG administration; (d) number of metaphase II oocytes retrieved; Figure S6: Forest plots of melatonin treatment on oocyte and embryo developmental outcomes. (a) oocyte survival rate; (b) oocyte maturation rate; (c) cleavage rate; (d) day 3 good-quality embryo rate; Figure S7: Forest plots of endogenous melatonin levels in women with polycystic ovary syndrome. (a) morning serum melatonin concentration; (b) nighttime serum melatonin concentration; Table S1: Database search strategies; Table S2: Characteristics of the included studies; Table S3: Quality assessment of observational studies using the Newcastle–Ottawa Scale (NOS); Table S4: Quality assessment of in vitro studies using the QUIN tool; Table S5: Unit-of-analysis characteristics and study-level results of in vitro studies; Table S6: Sensitivity analyses; Table S7. GRADE evidence profile for key outcomes; Table S8. Outcome-level data extraction of included studies.

## Abbreviations

The following abbreviations are used in this manuscript:

ART	Assisted reproductive technology
DOR	Diminished ovarian reserve
GRADE	Grading of Recommendations Assessment, Development and Evaluation
ICSI	Intracytoplasmic sperm injection
IUI	Intrauterine insemination
IVF	In vitro fertilization
MD	Mean difference
NOS	Newcastle–Ottawa Scale
PCOS	Polycystic ovary syndrome
POF	Premature ovarian failure
POI	Premature ovarian insufficiency
RCT	Randomized controlled trial
RoB 2	Risk of Bias 2
ROBINS-I	Risk Of Bias In Non-randomized Studies—of Interventions
