# Supplementary material for: Melatonin and Female Fertility: A Systematic Review and Meta-Analysis of Clinical Supplementation, In Vitro Experiments, and Observational Evidence
Source: Nutrients. 2026 Oct 19;18(20):3431. doi: 10.3390/nu18203431 (PMC13708123; doi:10.3390/nu18203431)
Supplement: Supplementary file 1 [file nutrients-18-03431-s001.zip › nutrients-4602290-supplementary.pdf]

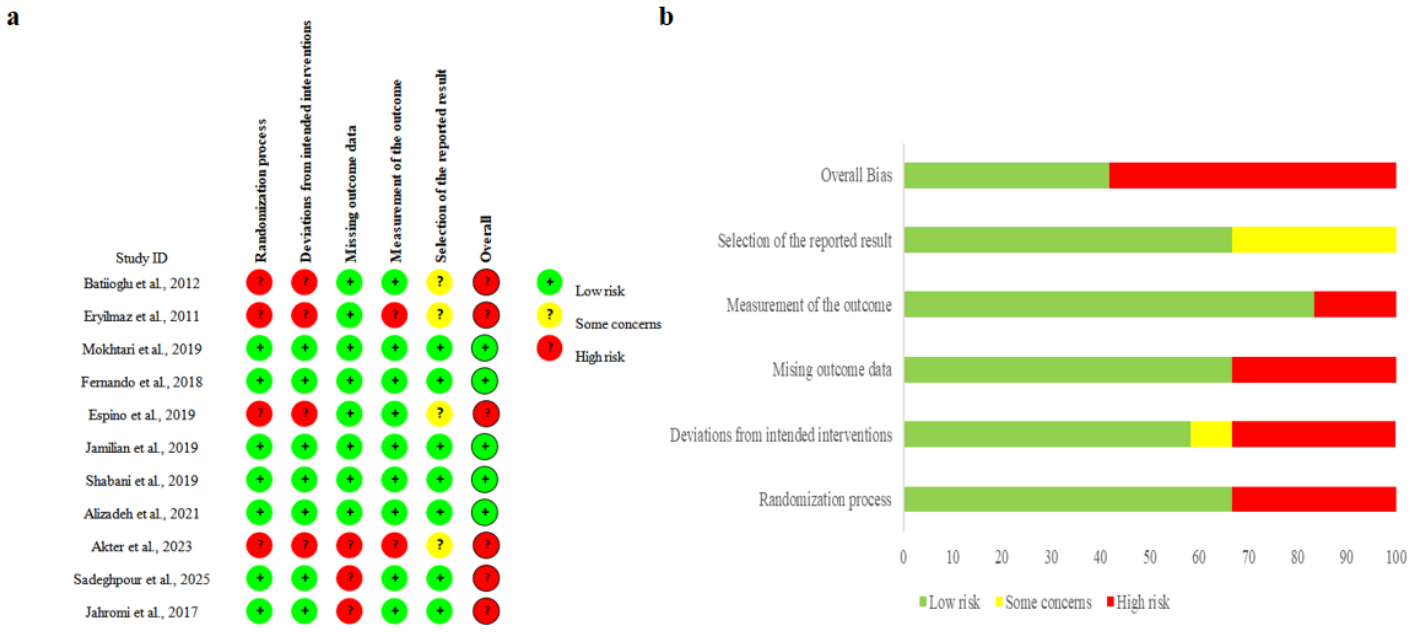

Figure S1. Risk of bias assessment of RCTs using RoB 2. (a) Risk-of-bias judgments for individual studies across domains; (b) summary of risk-of-bias judgments across domains[25-30,44-45,47-48,50].

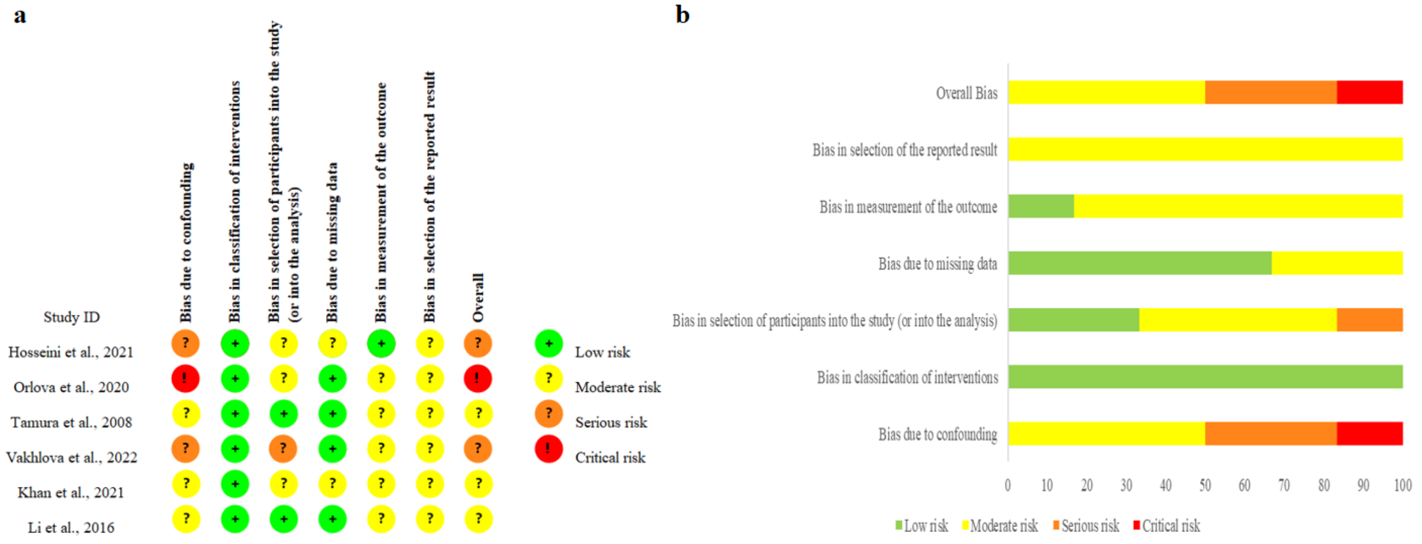

Figure S2. Risk of bias assessment of non-randomized studies using ROBINS-I. (a) Risk-of-bias judgments for individual studies across domains; (b) summary of risk-of-bias judgments across domains[23,31,49,51,52,62].

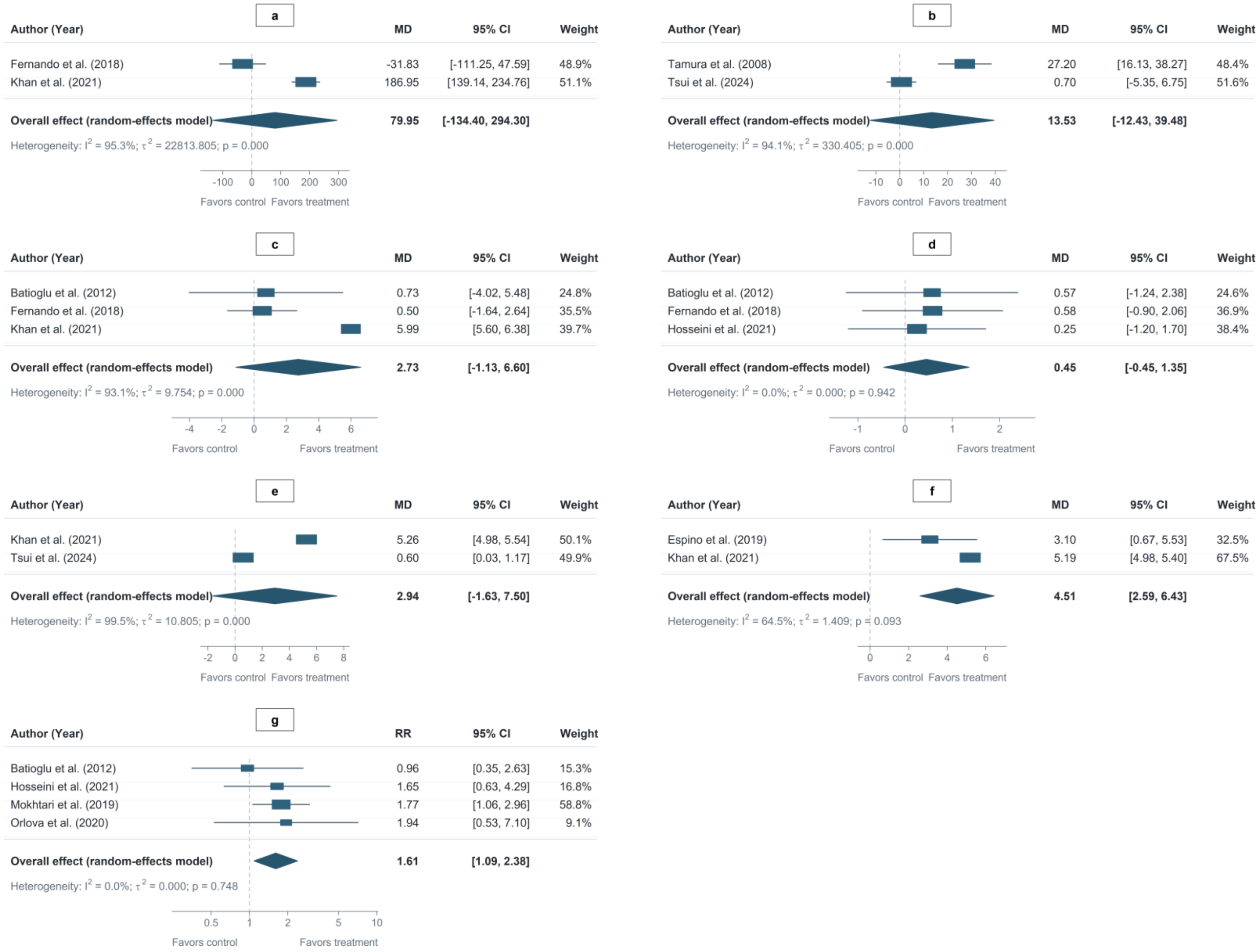

Figure S3. Forest plots of melatonin supplementation on ART outcomes. (a) follicular fluid estradiol concentration; (b) fertilization rate; (c) number of fertilized oocytes; (d) number of good-quality embryos; (e) number of day 3 good-quality embryos; (f) number of transferable embryos; (g) biochemical pregnancy rate[23-24,44-45,47,49-52].

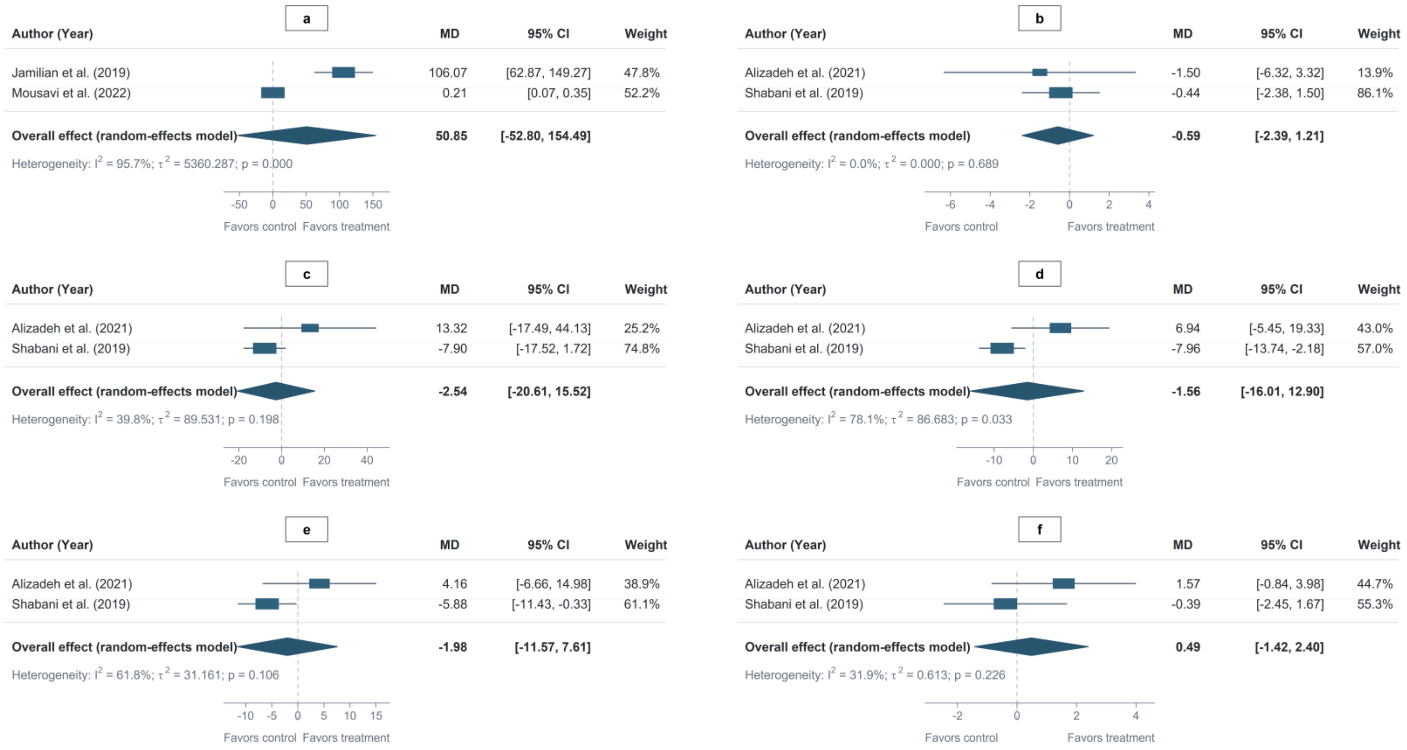

Figure S4. Forest plots of melatonin supplementation on PCOS-related outcomes. (a) total antioxidant capacity; (b) fasting blood glucose; (c) triglycerides; (d) total cholesterol; (e) low-density lipoprotein cholesterol; (f) high-density lipoprotein cholesterol[26-28,61].

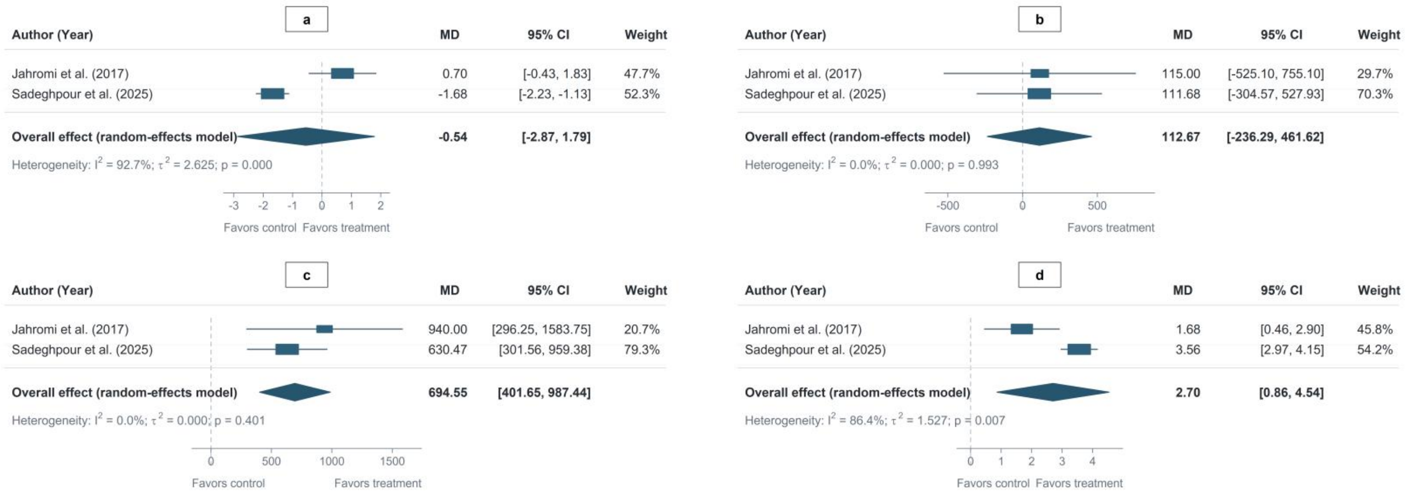

Figure S5. Forest plots of melatonin supplementation on DOR-related outcomes. (a) duration of ovarian stimulation; (b) total hMG dose; (c) serum estradiol concentration on the day of hCG administration; (d) number of metaphase II oocytes retrieved[29-30].

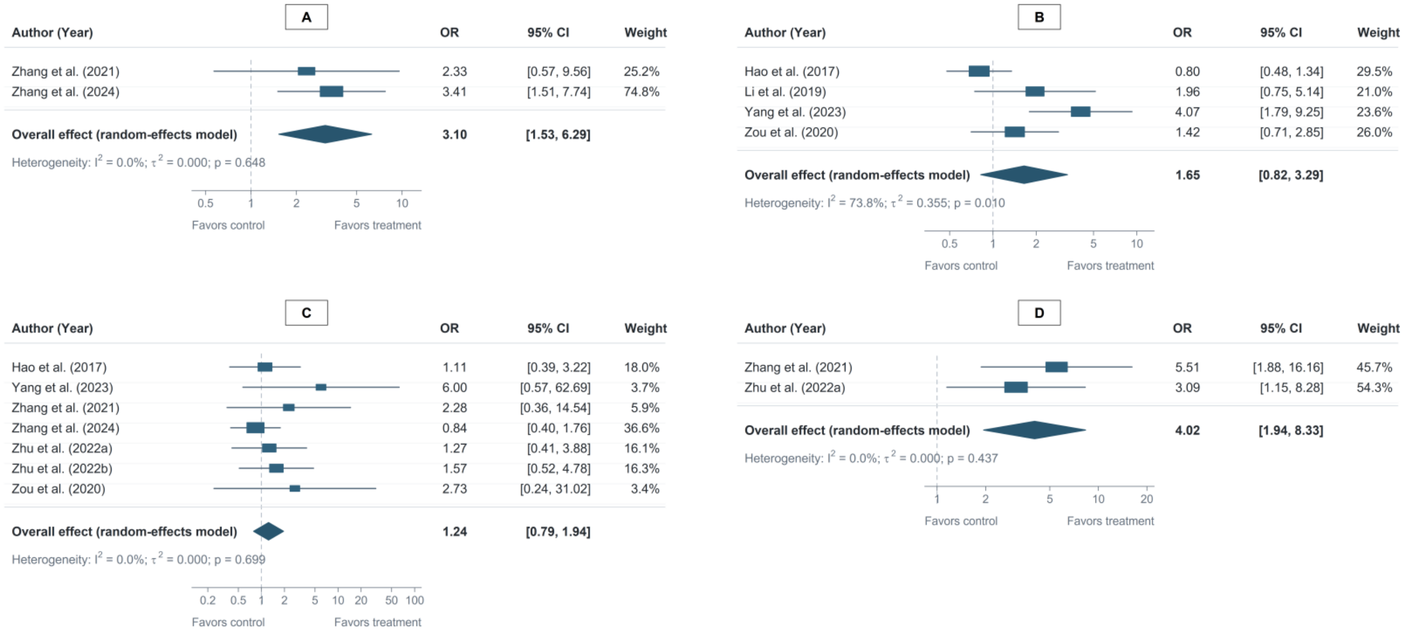

Figure S6. Forest plots of melatonin treatment on oocyte and embryo developmental outcomes. (a) oocyte survival rate; (b) oocyte maturation rate; (c) cleavage rate; (d) day 3 good-quality embryo rate[54-60,63].

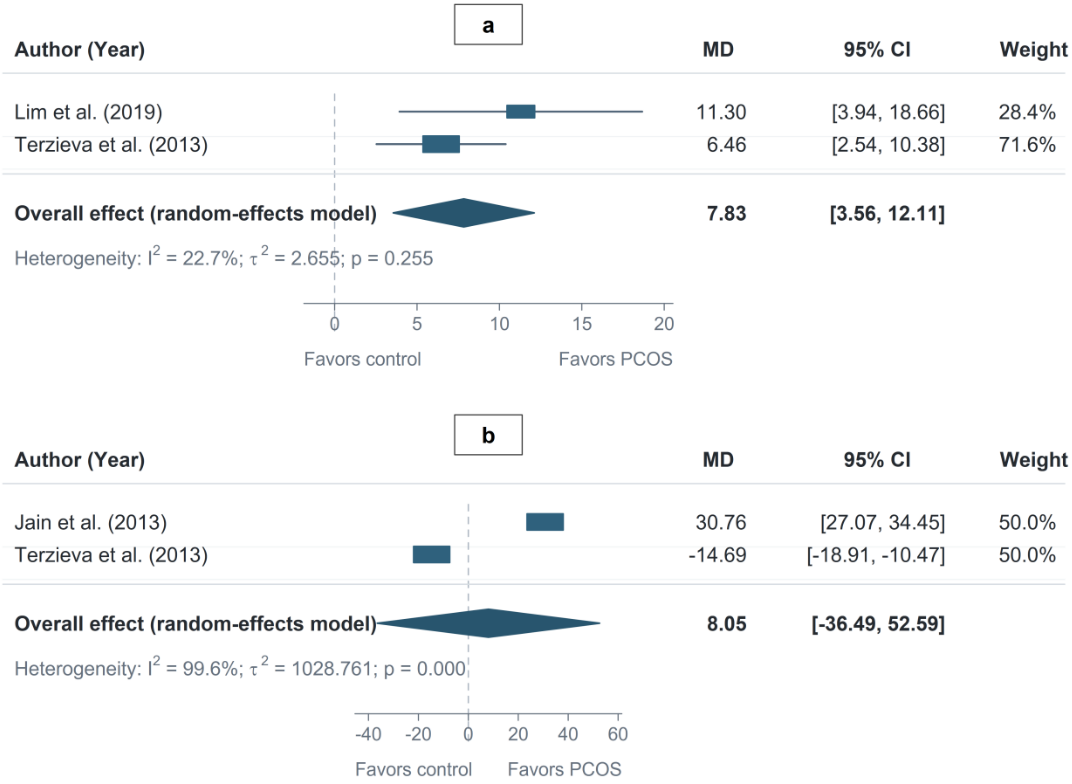

Figure S7. Forest plots of endogenous melatonin levels in women with polycystic ovary syndrome. (a) morning serum melatonin concentration; (b) nighttime serum melatonin concentration[64-66].

Table S1. Database search strategies.

Database	Search strategy
PubMed	#1 Melatonin:<br>melatonin OR melatonergic OR melatonina OR "5-methoxy-N-acetyltryptamine" OR "N-acetyl-5-methoxytryptamine"<br><br>#2 Reproduction-related terms:<br>"Assisted Reproductive Technique" OR "Assisted Reproductive Technology" OR "Reproductive Technique, Assisted" OR "Reproductive Technology, Assisted" OR ART OR "In Vitro Fertilization" OR IVF OR "Test-Tube Fertilization" OR "Intracytoplasmic Sperm Injection" OR ICSI OR "Artificial Insemination" OR "Intrauterine Insemination" OR IUI OR "polycystic ovary syndrome" OR PCOS OR "premature ovarian failure" OR "premature ovarian insufficiency" OR POF OR POI OR "diminished ovarian reserve" OR "decreased ovarian reserve" OR "poor ovarian reserve" OR "ovarian reserve decline"<br><br>#3 #1 AND #2
Web of Science	All Fields:<br>(melatonin OR melatonergic OR melatonine OR "N-acetyl-5-methoxytryptamine")<br>AND ("assisted reproductive technolog*" OR ART OR "in vitro fertilization" OR IVF OR ICSI OR "intracytoplasmic sperm injection" OR "intrauterine insemination" OR IUI OR "polycystic ovary syndrome" OR PCOS OR "premature ovarian insufficiency" OR POI OR POF OR "diminished ovarian reserve" OR "poor ovarian reserve")
Embase	#1 'melatonin'/exp OR melatonergic:ti,ab,kw OR melatonina:ti,ab,kw OR '5 methoxy n acetyltryptamine':ti,ab,kw OR 'n acetyl 5 methoxytryptamine':ti,ab,kw<br><br>#2 'infertility therapy'/exp OR 'assisted reproductive technique':ti,ab,kw OR 'assisted reproductive technology':ti,ab,kw OR 'reproductive technique, assisted':ti,ab,kw OR 'reproductive technology, assisted':ti,ab,kw OR art:ti,ab,kw OR 'in vitro fertilization':ti,ab,kw OR ivf:ti,ab,kw OR 'test-tube fertilization':ti,ab,kw OR 'intracytoplasmic sperm injection':ti,ab,kw OR icsi:ti,ab,kw OR 'artificial insemination':ti,ab,kw OR 'intrauterine insemination':ti,ab,kw OR iui:ti,ab,kw<br><br>#3 'ovary polycystic disease'/exp OR 'polycystic ovary syndrome':ti,ab,kw OR pcos:ti,ab,kw<br><br>#4 'premature ovarian failure'/exp OR 'premature ovarian insufficiency':ti,ab,kw OR pof:ti,ab,kw OR poi:ti,ab,kw OR 'diminished ovarian reserve':ti,ab,kw OR 'decreased ovarian reserve':ti,ab,kw OR 'poor ovarian reserve':ti,ab,kw OR 'ovarian reserve decline':ti,ab,kw<br><br>#5 #2 OR #3 OR #4<br>#6 #1 AND #5

Table S2. Characteristics of the included studies.

Study	Country	Study design	Study population/ material	Sample size	Melatonin intervention and comparator / measurement details	Main outcomes
Batioglu et al., 2012	Turkey	RCT	Women undergoing IVF-ET	85 (M/C: 40/45)	3 mg/day + IVF-ET vs IVF-ET	Mature oocytes; fertilization; embryo quality; clinical pregnancy; biochemical preganancy
Eryilmaz et al., 2011	Turkey	RCT	Women with sleep disorders undergoing IVF-ET	60 (M/C: 30/30)	3 mg/day + IVF-ET vs IVF-ET	Oocytes retrieved; mature oocytes
Hosseini et al., 2021	Iran	Non-randomized intervention study	Infertile couples undergoing IVF	90 (M/C: 45/45; analyzed: 35/42)	3 mg/day + COS vs COS	Mature oocytes; embryo quality; biochemical pregnancy; clinical pregnancy
Mokhtari et al., 2019	Iran	RCT	Women with PCOS undergoing IUI	198 (M/C: 98/100; analyzed: 94/100)	3 mg/day + IUI vs placebo + IUI	Biochemical pregnancy; clinical pregnancy
Orlova et al., 2020	Ukraine	Non-randomized intervention study	Women with tubal-peritoneal infertility undergoing IVF/ICSI	65 (M/C: 33/32)	3 mg/day + IVF/ICSI vs IVF/ICSI	Embryo quality; clinical pregnancy; live birth; biochemical pregnancy; miscarriage
Tamura et al., 2008	Japan	Non-randomized intervention study	Women with a low fertilization rate (<50%) and no pregnancy in a previous IVF-ET cycle	115 (M/C: 56/59)	3 mg/day + IVF-ET vs IVF-ET	Fertilization; clinical pregnancy
Vakhlova et al., 2022	Russia	Non-randomized intervention study	Infertile women undergoing IVF	84 (M/C: 39/45)	3 mg/day + IVF vs IVF	Blastocyst quality; clinical pregnancy
Tsui et al., 2024	China	Prospective cohort study	Women of advanced maternal age (35–45 years) undergoing IVF-FET	161 (M/C: 86/75)	2 mg/day + IVF-FET vs IVF-FET	Mature oocytes; clinical pregnancy; live birth; miscarriage
Khan et al., 2021	Pakistan	Non-randomized	Infertile women undergoing	242 (M/C: 145/97)	3 mg/day + IVF/ICSI	Fertilization; mature oocytes;

		intervention study	IVF/ICSI		vs IVF/ICSI	embryo quality
Fernando et al., 2018	Australia	RCT	Women undergoing IVF/ICSI	81 (M/C: 41/40)	4 mg/day + IVF/ICSI vs placebo + IVF/ICSI	Embryo quality; clinical pregnancy; live birth
Espino et al., 2019	Spain	RCT	Infertile women undergoing IVF/ICSI	20 (M/C: 10/10)	3 mg/day + IVF vs IVF	Embryo quality; clinical pregnancy
Bao et al., 2022	China	In vitro embryo culture	Human embryos	143 (M/C: 71/72)	100 nmol/L vs 0 nmol/L	Blastocyst formation; good-quality blastocysts
Zhu et al., 2022a[1]	China	Oocyte cryopreservation	Human oocytes	0-month treatment: 118 (M/C: 60/58)	1 nmol/L vs 0 nmol/L	Fertilization; cleavage; good-quality embryos; blastocyst formation
Zhang et al., 2024	China	Oocyte cryopreservation	Human oocytes	Autologous: 464 (M/C: 233/231)	1 nmol/L vs 0 nmol/L	Oocyte survival; fertilization; cleavage; good-quality embryos; good-quality blastocysts
Zhang et al., 2021	China	Oocyte cryopreservation	Human oocytes	106 (M/C: 51/55)	1 nmol/L vs 0 nmol/L	Oocyte survival; fertilization; cleavage; good-quality embryos; blastocyst formation
Zou et al., 2020	China	IVM	Human oocytes	193 (M/C: 105/88)	10^4 nmol/L vs 0 nmol/L	Oocyte maturation; fertilization; cleavage; blastocyst formation; good-quality blastocysts
Yang et al., 2023	China	IVM	Human oocytes	109 (M/C: 54/55)	10^4 nmol/L vs 0 nmol/L	Oocyte maturation; fertilization; cleavage; good-quality embryos; blastocyst formation; good-quality blastocysts
Li et al., 2019	China	IVM	Human oocytes	69 (M/C: 32/37)	10^4 nmol/L vs 0 nmol/L	Oocyte maturation
Hao et al., 2017	China	IVM	Human oocytes	294 (M/C: 159/135)	10^4 nmol/L vs 0 nmol/L	Oocyte maturation; fertilization; cleavage; blastocyst formation; good-quality blastocysts
Zhu et al., 2022b[1]	China	IVM	Human oocytes	118 (M/C: 60/58)	10^4 nmol/L vs 0 nmol/L	Fertilization; cleavage;

						blastocyst formation; good-quality blastocysts
Wei et al., 2013	China	IVM	Human oocytes	236 (M/C: 115/121)	10^4 nmol/L vs 0 nmol/L	Oocyte nuclear maturation
Jamilian et al., 2019	Iran	RCT	Women with PCOS	56 (M/C: 28/28)	10 mg/day vs placebo	Oxidative stress; hormonal outcomes; hirsutism
Shabani et al., 2019	Iran	RCT	Women with PCOS	58 (M/C: 29/29)	10 mg/day vs placebo	Sleep; psychological; metabolic outcomes
Alizadeh et al., 2021[2]	Iran	RCT	Women with PCOS	41 (M/C: 21/20)	6 mg/day vs placebo	Sleep; metabolic outcomes
Mousavi et al., 2022[2]	Iran	RCT	Women with PCOS	41 (M/C: 21/20)	6 mg/day vs placebo	Oxidative stress; clinical features
Akter et al., 2023	Bangladesh	RCT	Infertile women with PCOS	74 (M/C: 40/34)	3 mg/day + letrozole 5 mg/day vs letrozole 5 mg/day	Mature follicles; dominant follicle diameter; ovulation; clinical pregnancy
Lim et al., 2019	Singapore	Cross-sectional study	Women with PCOS / healthy controls	231 (PCOS/Control: 109/122)	07:30–08:30; overnight fasting; cycle days 2–5; ELISA (IBL)	Morning serum melatonin
Terzieva et al., 2013	Bulgaria	Case-control study	Women with PCOS / healthy controls	55 (PCOS/Control: 30/25)	03:00 and 08:00; cycle days 3–5; fasting status NR; ELISA (IBL-Hamburg)	Morning and nighttime serum melatonin
Abduljabbar et al., 2020	Iraq	Cross-sectional study	Women with PCOS / healthy controls	90 (PCOS/Control: 60/30)	07:00–08:00; cycle day 2; fasting status NR; assay method NR	Morning serum melatonin
Jain et al., 2013	India	Case-control study	Women with PCOS / healthy controls	100 (PCOS/Control: 50/50)	00:00–04:00; cycle day 2; fasting status NR; light/sleep conditions NR; competitive-	Nighttime serum melatonin

					inhibition ELISA (USCN)	
Li et al., 2022	China	Case-control study	Women with PCOS / healthy controls	71 (PCOS/Control: 35/36)	08:30–10:00 on oocyte-retrieval day; dark conditions; ^{125}I-radioimmunoassay	Follicular fluid melatonin
Luboshitzky et al., 2003	Israel	Case-control study	Women with PCOS / healthy controls	27 (PCOS/Control: 12/15)	24-h collection; menstrual-cycle timing NR; ELISA (IBL)	Urinary αMT6s
Shreeve et al., 2013	United Kingdom	Case-control study	Women with PCOS / healthy controls	33 (PCOS/Control: 15/18)	24-h collection in five predefined intervals; radioimmunoassay in duplicate	Nighttime urinary aMT6s
Sadeghpour et al., 2025	Iran	RCT	Women with DOR undergoing IVF/ICSI	68 (M/C: 35/33)	3 mg/day vs placebo	Mature oocytes; hCG-day serum estradiol; HMG dose; stimulation duration
Jahromi et al., 2017	Iran	RCT	Women with DOR undergoing IVF/ICSI	66 (M/C: 32/34)	3 mg/day vs placebo	Mature oocytes; hCG-day serum estradiol; HMG dose; stimulation duration
Li et al., 2016	China	Non-randomized intervention study	Women with POF	128 (M/C: 64/64)	1-3 mg/day vs placebo	Sex hormones; ROS; VEGF

1 Two independent studies published by the same first author in the same year were distinguished by the suffixes a and b.
2 Different outcomes reported from the same study.
3 M: Melatonin; C: Control.

Table S3. Quality assessment of observational studies using the Newcastle–Ottawa Scale (NOS).

**Study**	**Selection**				**Comparability**	**Outcome or exposure assessment**			**NOS score**
Cohort study	Representativeness of the exposed cohort	Selection of the non-exposed cohort	Ascertainment of exposure	Demonstration that outcome of interest was not present at start of study	Comparability of cohorts on the basis of the design or analysis	Assessment of outcome	Follow-up long enough	Adequacy of follow-up	
Tsui et al., 2024	★	★	★	★	★	★	★	0	7/9
Case-control study	Adequate case definition	Representativeness of cases	Selection of controls	Definition of controls	Comparability of cohorts on the basis of the design or analysis	Ascertainment of exposure	Same method of ascertainment	Non-response rate	
Terzieva et al., 2013	★	0	0	0	★	★	★	0	4/9
Jain et al., 2013	★	0	★	★	★★	★	★	0	7/9
Li et al., 2022	★	0	★	★	★★	★	★	0	7/9
Luboshitzky et al., 2003	★	0	0	★	★	★	★	0	5/9
Shreeve et al., 2013	★	0	0	0	0	★	★	0	3/9
Cross-sectional study	Representativeness of sample	Sample size justification	Non-respondents	Ascertainment of exposure	Comparability of cohorts on the basis of the design or analysis	Assessment of outcome	Statistical test		
Lim et al., 2019	★	0	0	★★	★★	★★	★		8/10
Abduljabbar et al., 2020	★	0	0	★	★	★★	★		6/10

* Each star (★) represents one point. The original NOS used for cohort and case-control studies has a maximum score of 9 stars (4 for Selection, 2 for Comparability, and 3 for Outcome), whereas the adapted NOS described by Herzog et al. (2013) used for cross-sectional studies has a maximum score of 10 stars (5 for Selection, 2 for Comparability, and 3 for Outcome).

Table S4. Quality assessment of in vitro studies using the QUIN tool.

Study ID	Aims/ Objectives	Sample size calculation	Sampling technique	Compariso n group	Methodology	Operators	Randomi zation	Outcome measurement	Outcome assessors	Blinding	Statistical analysis	Results presentation	Total (/24)	Final score (%)	Risk of bias
Bao et al., 2022	2	0	2	2	2	2	1	2	2	0	2	2	19	79.17	Low
Zhu et al., 2022a	2	0	2	2	2	0	0	2	0	0	2	2	14	58.33	Medium
Zhang et al., 2024	2	0	2	2	2	0	0	2	0	0	2	2	14	58.33	Medium
Zhang et al., 2021	2	0	2	2	2	0	1	2	0	0	2	2	15	62.50	Medium
Zou et al., 2020	2	0	1	2	2	0	1	2	0	0	2	2	14	58.33	Medium
Yang et al., 2023	2	0	2	2	2	1	1	2	1	0	2	2	17	70.83	Low
Li et al., 2019	2	0	1	2	2	0	1	2	0	0	2	2	14	58.33	Medium
Hao et al., 2017	2	0	2	2	2	0	1	2	0	0	2	2	15	62.50	Medium
Zhu et al., 2022b	2	0	2	2	2	1	1	2	1	0	2	2	17	70.83	Low
Wei et al., 2013	2	0	2	2	2	1	1	2	1	0	2	2	17	70.83	Low

Table S5. Unit-of-analysis characteristics and study-level results of the included in vitro studies.

Study	No. of women/donors	No. of oocytes/embryos	Allocation structure	Sibling oocytes/embryos allocated across conditions	Clustering/pairing adjustment	Outcome	OR (95%CI)
Zhang et al., 2024	146 (donor/autologous: 108/38)	464 (M/C: 233/231)	Voluntary patient choice	No	No adjustment reported	Oocyte survival rate	3.41 (1.51 - 7.74)
Zhang et al., 2024	146 (donor/autologous: 108/38)	464 (M/C: 233/231)	Voluntary patient choice	No	No adjustment reported	Fertilization rate	1.96 (1.06 - 3.64)
Zhang et al., 2024	146 (donor/autologous: 108/38)	464 (M/C: 233/231)	Voluntary patient choice	No	No adjustment reported	Cleavage rate	0.84 (0.40 - 1.76)
Zhang et al., 2024	146 (donor/autologous: 108/38)	464 (M/C: 233/231)	Voluntary patient choice	No	No adjustment reported	Good-quality embryo rate	2.23 (1.45 - 3.42)
Zhang et al., 2024	146 (donor/autologous: 108/38)	464 (M/C: 233/231)	Voluntary patient choice	No	No adjustment reported	Blastocyst formation rate	1.23 (0.80 - 1.88)
Zhang et al., 2024	146 (donor/autologous: 108/38)	464 (M/C: 233/231)	Voluntary patient choice	No	No adjustment reported	Good-quality blastocyst rate	1.84 (1.13 - 2.99)
Zhu et al., 2022a	198 (screening experiment)	118 (M/C: 60/58)	Allocation method unclear (screening experiment)	Unclear	No adjustment reported	Fertilization rate	3.06 (1.29 - 7.23)
Zhu et al., 2022a	198 (screening experiment)	118 (M/C: 60/58)	Allocation method unclear (screening experiment)	Unclear	No adjustment reported	Cleavage rate	1.27 (0.41 - 3.88)
Zhu et al., 2022a	198 (screening experiment)	118 (M/C: 60/58)	Allocation method unclear (screening experiment)	Unclear	No adjustment reported	Day 3 good-quality embryo rate	3.09 (1.15 - 8.28)
Zhu et al., 2022a	198 (screening experiment)	118 (M/C: 60/58)	Allocation method unclear (screening experiment)	Unclear	No adjustment reported	Blastocyst formation rate	3.26 (1.04 - 10.24)
Li et al., 2019	NR	69 (M/C: 32/37)	Random allocation	Unclear	No adjustment	Oocyte maturation rate	1.96 (0.75 - 5.14)

			of oocytes		reported		
Hao et al., 2017	200	294 (M/C: 159/135)	Random allocation of oocytes	Unclear	No adjustment reported	Oocyte maturation rate	0.80 (0.48 - 1.34)
Hao et al., 2017	200	294 (M/C: 159/135)	Random allocation of oocytes	Unclear	No adjustment reported	Fertilization rate	2.41 (1.10 - 5.29)
Hao et al., 2017	200	294 (M/C: 159/135)	Random allocation of oocytes	Unclear	No adjustment reported	Cleavage rate	1.11 (0.39 - 3.22)
Hao et al., 2017	200	294 (M/C: 159/135)	Random allocation of oocytes	Unclear	No adjustment reported	Blastocyst formation rate	1.64 (0.78 - 3.42)
Hao et al., 2017	200	294 (M/C: 159/135)	Random allocation of oocytes	Unclear	No adjustment reported	Good-quality blastocyst rate	6.29 (1.38 - 28.65)
Zhu et al., 2022b	140	119 (M/C: 58/61)	Random allocation	Unclear	No adjustment reported	Fertilization rate	1.26 (0.44 - 3.64)
Zhu et al., 2022b	140	119 (M/C: 58/61)	Random allocation	Unclear	No adjustment reported	Cleavage rate	1.57 (0.52 - 4.78)
Zhu et al., 2022b	140	119 (M/C: 58/61)	Random allocation	Unclear	No adjustment reported	Blastocyst formation rate	0.64 (0.19 - 2.21)
Zhu et al., 2022b	140	119 (M/C: 58/61)	Random allocation	Unclear	No adjustment reported	Good-quality blastocyst rate	0.95 (0.13 - 7.09)
Bao et al., 2022	NR (Experiment II)	143 (M/C: 71/72)	Random allocation of warmed embryos	Unclear	No adjustment reported	Blastocyst formation rate	2.04 (1.01 - 4.13)
Bao et al., 2022	NR (Experiment II)	143 (M/C: 71/72)	Random allocation of warmed embryos	Unclear	No adjustment reported	Good-quality blastocyst rate	1.20 (0.33 - 4.34)
Zhang et al., 2021	236	106 (M/C: 51/55)	Random allocation of oocytes	Unclear	No adjustment reported	Oocyte survival rate	2.33 (0.57 - 9.56)
Zhang et al.,	236	106 (M/C: 51/55)	Random allocation	Unclear	No adjustment	Fertilization rate	5.16 (1.73 - 15.42)

2021			of oocytes		reported		
Zhang et al., 2021	236	106 (M/C: 51/55)	Random allocation of oocytes	Unclear	No adjustment reported	Cleavage rate	2.28 (0.36 - 14.54)
Zhang et al., 2021	236	106 (M/C: 51/55)	Random allocation of oocytes	Unclear	No adjustment reported	Day 3 good-quality embryo rate	5.51 (1.88 - 16.16)
Zhang et al., 2021	236	106 (M/C: 51/55)	Random allocation of oocytes	Unclear	No adjustment reported	Blastocyst formation rate	5.12 (1.32 - 19.84)
Yang et al., 2023	255	109 (M/C: 54/55)	Random allocation within age group	Yes	No adjustment reported	Oocyte maturation rate	4.07 (1.79 - 9.25)
Yang et al., 2023	255	109 (M/C: 54/55)	Random allocation within age group	Yes	No adjustment reported	Fertilization rate	1.03 (0.32 - 3.32)
Yang et al., 2023	255	109 (M/C: 54/55)	Random allocation within age group	Yes	No adjustment reported	Cleavage rate	6.00 (0.57 - 62.69)
Yang et al., 2023	255	109 (M/C: 54/55)	Random allocation within age group	Yes	No adjustment reported	Good-quality embryo rate	2.00 (0.55 - 7.27)
Yang et al., 2023	255	109 (M/C: 54/55)	Random allocation within age group	Yes	No adjustment reported	Blastocyst formation rate	7.43 (1.42 - 38.78)
Yang et al., 2023	255	109 (M/C: 54/55)	Random allocation within age group	Yes	No adjustment reported	Good-quality blastocyst rate	8.22 (0.43 - 156.50)
Zou et al., 2020	NR (main experiment)	193 (M/C: 105/88)	Random allocation	Unclear	No adjustment reported	Oocyte maturation rate	1.42 (0.71 - 2.85)
Zou et al., 2020	NR (main experiment)	193 (M/C: 105/88)	Random allocation	Unclear	No adjustment reported	Fertilization rate	1.19 (0.55 - 2.55)
Zou et al., 2020	NR (main experiment)	193 (M/C: 105/88)	Random allocation	Unclear	No adjustment reported	Cleavage rate	2.73 (0.24 - 31.02)
Zou et al.,	NR (main experiment)	193 (M/C: 105/88)	Random allocation	Unclear	No adjustment	Blastocyst formation rate	2.99 (1.33 - 6.72)

2020					reported		
Zou et al., 2020	NR (main experiment)	193 (M/C: 105/88)	Random allocation	Unclear	No adjustment reported	Good-quality blastocyst rate	19.00 (2.45 - 147.63)
Wei et al., 2013	146	236 (M/C: 115/121)	Random allocation	Unclear	No adjustment reported	Oocyte nuclear maturation	Not pooled

* NR: Not reported; M: Melatonin; C: Control

Table S6. Sensitivity analyses.

Outcome	Excluded study	Effect estimate (95% CI)	P value	I^2
**A. Leave-one-out sensitivity analysis**				
**Number of oocytes retrieved**	None	MD 1.24 (-0.004 - 2.49)	0.051	46.8%
	Batioglu et al., 2012	MD 1.26 (-0.05 - 2.56)	0.059	54.4%
	Eryilmaz et al., 2011	MD 0.83 (0.15 - 1.51)	0.016	0.0%
	Hosseini et al., 2021	MD 1.52 (0.17 - 2.87)	0.027	47.1%
	Orlova et al., 2020	MD 1.52 (0.20 - 2.84)	0.024	46.5%
	Tsui et al., 2024	MD 1.17 (-0.42 - 2.75)	0.149	50.9%
	Khan et al., 2021	MD 1.40 (-0.22 - 3.03)	0.090	50.3%
	Fernando et al., 2018	MD 1.32 (-0.04 - 2.69)	0.058	53.9%
	Espino et al., 2019	MD 1.06 (-0.26 - 2.38)	0.115	48.7%
**Number of mature oocytes**	None	MD 2.62 (0.36 - 4.88)	0.023	95.2%
	Batioglu et al., 2012	MD 2.96 (0.24 - 5.67)	0.033	95.6%
	Eryılmaz et al., 2011	MD 1.16 (−0.50 - 4.81)	0.111	96.4%
	Hosseini et al., 2021	MD 3.30 (0.99 - 5.61)	0.005	94.8%
	Tsui et al., 2024	MD 2.90 (0.06 - 5.73)	0.045	93.1%
	Khan et al., 2021	MD 1.69 (−0.17 - 3.55)	0.074	67.8%
**Clinical pregnancy rate**	None	RR 1.55 (1.23 - 1.94)	<0.001	0.0%
	Batioglu et al., 2012	RR 1.65 (1.27 - 2.14)	<0.001	0.0%
	Hosseini et al., 2021	RR 1.53 (1.22 - 1.93)	<0.001	0.0%
	Mokhtari et al., 2019	RR 1.50 (1.17 - 1.92)	0.002	0.0%
	Orlova et al., 2020	RR 1.59 (1.23 - 2.04)	<0.001	0.0%
	Tamura et al., 2008	RR 1.53 (1.20 - 1.93)	<0.001	0.0%
	Tsui et al., 2024	RR 1.52 (1.17 - 1.99)	0.002	0.0%
	Fernando et al., 2018	RR 1.53 (1.21 - 1.94)	<0.001	0.0%
	Espino et al., 2019	RR 1.55 (1.23 - 1.95)	<0.001	0.0%
**Blastocyst formation rate**	None	OR 2.00 (1.33 - 3.03)	<0.001	48.4%
	Bao et al., 2022	OR 2.07 (1.23 - 3.47)	0.006	55.1%

Outcome	Excluded study	Effect estimate (95% CI)	P value	I ²
	Zhu et al., 2022a	OR 1.91 (1.23 - 2.96)	0.004	51.5%
	Zhang et al., 2024	OR 2.27 (1.54 - 3.35)	<0.001	33.8%
	Zhang et al., 2021	OR 1.84 (1.23 - 2.73)	0.003	45.7%
	Zou et al., 2020	OR 1.88 (1.20 - 2.93)	0.006	48.5%
	Yang et al., 2023	OR 1.84 (1.24 - 2.71)	0.002	43.1%
	Hao et al., 2017	OR 2.15 (1.29 - 3.58)	0.003	55.7%
	Zhu et al., 2022b	OR 2.21 (1.44 - 3.37)	<0.001	45.0%
**Fertilization rate**	None	OR 1.97 (1.40 - 2.76)	<0.001	22.8%
	Zhu et al., 2022a	OR 1.83 (1.28 - 2.62)	<0.001	24.2%
	Zhang et al., 2024	OR 1.97 (1.24 - 3.15)	0.004	35.6%
	Zhang et al., 2021	OR 1.79 (1.28 - 2.51)	<0.001	0.0%
	Zou et al., 2020	OR 2.19 (1.54 - 3.11)	<0.001	12.9%
	Yang et al., 2023	OR 2.08 (1.46 - 2.96)	<0.001	23.2%
	Hao et al., 2017	OR 1.89 (1.24 - 2.88)	0.003	32.9%
	Zhu et al., 2022b	OR 2.07 (1.42 - 3.00)	<0.001	28.9%
**B. Sensitivity analysis excluding studies at high, serious, or critical risk of bias**				
**Number of oocytes retrieved**	None	MD 1.24 (-0.004 - 2.49)	0.051	46.8%
	Batioglu et al., 2012; Eryilmaz et al., 2011; Hosseini et al., 2021; Orlova et al., 2020; Espino et al., 2019	MD 1.00 (0.23 - 1.76)	0.011	0.0%
**Number of mature oocytes**	None	MD 2.62 (0.36 - 4.88)	0.023	95.2%
	Batioglu et al., 2012; Eryilmaz et al., 2011; Hosseini et al., 2021	MD 3.65 (-0.47 - 7.78)	0.083	97.6%
**Number of fertilized oocytes**	None	MD 2.73 (-1.13 - 6.60)	0.165	93.1%
	Batioglu et al., 2012	MD 3.35 (-2.03 - 8.73)	0.222	95.9%
**Clinical pregnancy rate**	None	RR 1.55 (1.23 - 1.94)	<0.001	0.0%

Outcome	Excluded study	Effect estimate (95% CI)	P value	I ²
	Batioglu et al., 2012;<br>Orlova et al., 2020;<br>Hosseini et al., 2021;<br>Espino et al., 2019	RR 1.73 (1.27 - 2.36)	<0.001	0.0%

Table S7. GRADE evidence profile for key outcomes.

Certainty assessment							№ of patients		Effect		Certainty	Importance
№ of studies	Study design	Risk of bias	Inconsistency	Indirectness	Imprecision	Other considerations	Intervention	Control	Relative (95% CI)	Absolute (95% CI)		
Number of oocytes retrieved												
4	randomised trials	serious[a]	not serious	not serious	serious[b]	none	118	121	-	MD 3.07 higher (0.97 higher to 5.17 higher)	Low	CRITICAL
Number of oocytes retrieved												
4	non-randomised studies	serious[a]	not serious	not serious	not serious	none	299	246	-	MD 0.75 higher (0.05 higher to 1.45 higher)	Very low	CRITICAL
Clinical pregnancy rate												
4	randomised trials	serious[a]	not serious	not serious	not serious	none	60/185 (32.4%)	41/195 (21.0%)	RR 1.51 (1.08 to 2.10)	107 more per 1,000 (from 17 more to 231 more)	Moderate	CRITICAL
Clinical pregnancy rate												
4	non-randomised studies	serious[a]	not serious	not serious	not serious	none	70/208 (33.7%)	41/207 (19.8%)	RR 1.59 (1.15 to 2.18)	117 more per 1,000 (from 30 more to 234 more)	Very low	CRITICAL
Live birth rate												
2	Mixed designs (1RCT + 1 cohort)	not serious	not serious	not serious	serious[c]	none	39/127 (30.7%)	22/115 (19.1%)	RR 1.59 (1.01 to 2.51)	113 more per 1,000 (from 2 more to 289 more)	Low	CRITICAL
Blastocyst formation rate												
8	non-randomised studies	not serious[d]	serious[e]	not serious	not serious	none	226/576 (39.2%)	127/471 (27.0%)	OR 2.00 (1.33 to 3.03)	155 more per 1,000 (from 60 more to 258 more)	Very low	CRITICAL
Fertilization rate												
7	non-randomised studies	not serious[d]	not serious	not serious	not serious	none	551/630 (87.5%)	443/565 (78.4%)	OR 1.97 (1.40 to 2.76)	93 more per 1,000 (from 52 more to 125 more)	Very low	CRITICAL

a. Most included studies were assessed as having moderate to high risk of bias across one or more domains.
b. Optimal information size (OIS) (for continuous outcomes, OIS = 400 participants) not met.
c. The confidence interval was wide and its lower bound was close to the null effect.
d. Most included in vitro studies did not account for within-participant correlation among oocytes or embryos.
e. Moderate heterogeneity was observed ($I^2 = 48.4\%$), but prespecified subgroup analyses did not identify a clear source of heterogeneity.

Table S8. Outcome-level data extraction of included studies

Study	Group/comparison	Outcome	Sample size	Outcome Data	Source location
Batioglu et al., 2012	Melatonin 3 mg/day	Number of oocytes retrieved	40	15.33 [4, 27]	Table 1
	Control	Number of oocytes retrieved	45	14.27 [4, 25]	Table 1
	Melatonin 3 mg/day	Number of mature oocytes	40	12±6	Table 1
	Control	Number of mature oocytes	45	10.9±4	Table 1
	Melatonin 3 mg/day	Number of fertilized oocytes	40	9 [2, 17]	Table 1
	Control	Number of fertilized oocytes	45	7.8 [2, 16]	Table 1
	Melatonin 3 mg/day	Number of good-quality embryos	40	3.2 [0, 6]	Table 1
	Control	Number of good-quality embryos	45	2.5 [0, 5]	Table 1
	Melatonin 3 mg/day	Clinical pregnancy rate (denominator: women completing IVF-ET)	40	20	Table 1
	Control	Clinical pregnancy rate (denominator: women completing IVF-ET)	45	18	Table 1
	Melatonin 3 mg/day	Biochemical pregnancy rate (denominator: women completing IVF-ET)	40	6	Table 1
	Control	Biochemical pregnancy rate (denominator: women completing IVF-ET)	45	7	Table 1
Eryilmaz et al., 2011	Melatonin 3 mg/day	Number of oocytes retrieved	30	11.5±6.3	Table 2
	Control	Number of oocytes retrieved	30	6.9±3.8	Table 2
	Melatonin 3 mg/day	Number of mature oocytes	30	9±5.6	Table 2
	Control	Number of mature oocytes	30	4.4±3.3	Table 2
Hosseini et al., 2021	Melatonin 3 mg/day	Number of oocytes retrieved	35	9.36±5.37	Table 3
	Control	Number of oocytes retrieved	42	9.8±4.72	Table 3
	Melatonin 3 mg/day	Number of mature oocytes	35	7.8±5.13	Table 3
	Control	Number of mature oocytes	42	8.09±4.88	Table 3

	Melatonin 3 mg/day	Total number of embryos	35	67.27±174.92	Table 3
	Control	Total number of embryos	42	19.35±84.73	Table 3
	Melatonin 3 mg/day	Number of good-quality embryos	33	4.66±3.35	Table 3
	Control	Number of good-quality embryos	41	4.41±2.93	Table 3
	Melatonin 3 mg/day	Biochemical pregnancy rate (denominator: analyzed participants)	34	8	Table 3
	Control	Biochemical pregnancy rate (denominator: analyzed participants)	42	6	Table 3
	Melatonin 3 mg/day	Clinical pregnancy rate (denominator: analyzed participants)	33	5	Table 3
	Control	Clinical pregnancy rate (denominator: analyzed participants)	41	3	Table 3
Mokhtari et al., 2019	Melatonin 3 mg/day	Biochemical pregnancy rate (denominator: analyzed participants/IUI cycles)	94	30	Table 2
	Control	Biochemical pregnancy rate (denominator: analyzed participants/IUI cycles)	100	18	Table 2
	Melatonin 3 mg/day	Clinical pregnancy rate (denominator: analyzed participants/IUI cycles)	94	26	Table 2
	Control	Clinical pregnancy rate (denominator: analyzed participants/IUI cycles)	100	15	Table 2
Orlova et al., 2020	Melatonin 3 mg/day	Number of oocytes retrieved	33	12.8±4.77	Table 1
	Control	Number of oocytes retrieved	32	13.5±5.71	Table 1
	Melatonin 3 mg/day	Fertilization rate	33	85.7%	Table 1
	Control	Fertilization rate	32	88.4%	Table 1
	Melatonin 3 mg/day	Number of transferable embryos	33	1.8±1.32	Table 1
	Control	Number of transferable embryos	32	1.08±1.36	Table 1
	Melatonin 3 mg/day	Clinical pregnancy rate (denominator: included women)	33	51.51%	Result section, p.2375
	Control	Clinical pregnancy rate (denominator: included women)	32	37.51%	Result section, p.2375
	Melatonin 3 mg/day	Biochemical pregnancy rate (denominator: included women)	33	18.18%	Result section, p.2375

	Control	Biochemical pregnancy rate (denominator: included women)	32	9%	Result section, p.2375
	Melatonin 3 mg/day	Miscarriage rate (denominator: included women)	33	3%	Result section, p.2375
	Control	Miscarriage rate (denominator: included women)	32	12.5%	Result section, p.2375
Tamura et al., 2008	Melatonin 3 mg/day	Fertilization rate (%)	56	50±38	Table 1
	Control	Fertilization rate (%)	59	22.8±19	Table 1
	Melatonin 3 mg/day	Clinical pregnancy rate (denominator: IVF-ET cycles)	56	11	Table 1
	Control	Clinical pregnancy rate (denominator: IVF-ET cycles)	59	6	Table 1
Vakhlova et al., 2022	Melatonin 3 mg/day	Pregnancy rate in fresh cycles (denominator: embryo-transfer cycles)	14	4	Result section, p.7
	Control	Pregnancy rate in fresh cycles (denominator: embryo-transfer cycles)	20	7	Result section, p.7
	Melatonin 3 mg/day	Pregnancy rate in frozen cycles (denominator: embryo-transfer cycles)	24	16	Table 7
	Control	Pregnancy rate in frozen cycles (denominator: embryo-transfer cycles)	22	6	Table 7
Tsui et al., 2024	Melatonin 2 mg/day	Number of oocytes retrieved	86	8.8±5.3	Table 1
	Control	Number of oocytes retrieved	75	7.1±4.1	Table 1
	Melatonin 2 mg/day	Number of mature oocytes	86	7±4.8	Table 1
	Control	Number of mature oocytes	75	5.5±3.2	Table 1
	Melatonin 2 mg/day	Fertilization rate (%)	86	84±18	Table 1
	Control	Fertilization rate (%)	75	83.3±20.8	Table 1
	Melatonin 2 mg/day	Number of day-3 embryos	86	5.5±3.9	Table 1
	Control	Number of day-3 embryos	75	4.2±2.8	Table 1
	Melatonin 2 mg/day	Number of good-quality day-3 embryos	86	2.3±1.9	Table 1
	Control	Number of good-quality day-3 embryos	75	1.7±1.8	Table 1

	Melatonin 2 mg/day	Clinical pregnancy rate (denominator: participants)	86	43%	Figure 1
	Control	Clinical pregnancy rate (denominator: participants)	75	26.70%	Figure 1
	Melatonin 2 mg/day	Live birth rate (denominator: participants)	86	32.60%	Figure 1
	Control	Live birth rate (denominator: participants)	75	21.60%	Figure 1
	Melatonin 2 mg/day	Miscarriage rate (denominator: clinical pregnancies)	37	24.30%	Supplementary material
	Control	Miscarriage rate (denominator: clinical pregnancies)	20	20%	Supplementary material
Khan2021	Melatonin 3 mg/day	Number of oocytes retrieved	145	14.09±2.9	Table 2
	Control	Number of oocytes retrieved	97	13.34±3.88	Table 2
	Melatonin 3 mg/day	Number of mature oocytes	145	10.12±1.11	Table 2
	Control	Number of mature oocytes	97	4.41±0.94	Table 2
	Melatonin 3 mg/day	Number of fertilized oocytes	145	9.98±2.12	Table 2
	Control	Number of fertilized oocytes	97	3.99±0.95	Table 2
	Melatonin 3 mg/day	Number of good-quality day-3 embryos	145	6.32±1.43	Table 2
	Control	Number of good-quality day-3 embryos	97	1.06±0.76	Table 2
	Melatonin 3 mg/day	Number of transferable embryos	145	6.22±0.98	Table 2
	Control	Number of transferable embryos	97	1.03±0.65	Table 2
	Melatonin 3 mg/day	Follicular estradiol concentration (ng/mL)	145	598.15±258.74	Table 1
	Control	Follicular estradiol concentration (ng/mL)	97	411.2±113.69	Table 1
Fernando2018	Melatonin 4 mg/day	Follicular estradiol concentration (pmol/L)	38	705000 [425000, 1.19e+06]	Table 2
	Control	Follicular estradiol concentration (pmol/L)	36	851000 [529500, 1.29e+06]	Table 2
	Melatonin 4 mg/day	Number of oocytes retrieved	41	9 [0, 16]	Table 3
	Control	Number of oocytes retrieved	40	8 [3.25, 13]	Table 3

	Melatonin 4 mg/day	Number of fertilized oocytes	41	4 [0, 7.5]	Table 3
	Control	Number of fertilized oocytes	40	3 [1, 6]	Table 3
	Melatonin 4 mg/day	Number of good-quality embryos	41	2 [0, 5]	Table 3
	Control	Number of good-quality embryos	40	1.5 [0, 3.75]	Table 3
	Melatonin 4 mg/day	Clinical pregnancy rate (denominator: randomized women, ITT)	41	11	Table 4
	Control	Clinical pregnancy rate (denominator: randomized women, ITT)	40	6	Table 4
	Melatonin 4 mg/day	Live birth rate (denominator: randomized women, ITT)	41	11	Table 4
	Control	Live birth rate (denominator: randomized women, ITT)	40	6	Table 4
Espino2019	Melatonin 3 mg/day	Number of oocytes retrieved	10	10 (6.7 - 13.3)	Table 2
	Control	Number of oocytes retrieved	10	6.8 (4.4 - 9.2)	Table 2
	Melatonin 3 mg/day	Number of transferable embryos	10	5.1 (2.8 - 7.4)	Table 2
	Control	Number of transferable embryos	10	2 (0.4 - 3.6)	Table 2
	Melatonin 3 mg/day	Clinical pregnancy rate (denominator: initiated IVF cycles)	10	3	Table 2
	Control	Clinical pregnancy rate (denominator: initiated IVF cycles)	10	2	Table 2
Bao et al., 2022	Melatonin 100 nmol/L	Number of good-quality day-3 embryos	135	40	Table 4
	Control	Number of good-quality day-3 embryos	442	86	Table 4
	Melatonin 100 nmol/L	Clinical pregnancy rate	24	6	Table 4
	Control	Clinical pregnancy rate	41	7	Table 4
	Melatonin 100 nmol/L	Number of blastocysts	71	30	Table 5
	Control	Number of blastocysts	72	19	Table 5
	Melatonin 100 nmol/L	Number of good-quality blastocysts	30	9	Table 5
	Control	Number of good-quality blastocysts	19	5	Table 5
Zhu et al.,	Melatonin 1 nmol/L (0-	Fertilization rate	60	50	Table 1

2022a	month cryopreservation)				
	Control	Fertilization rate	58	36	Table 1
	Melatonin 1 nmol/L (0-month cryopreservation)	Cleavage rate	50	42	Table 1
	Control	Cleavage rate	36	29	Table 1
	Melatonin 1 nmol/L (0-month cryopreservation)	Number of good-quality day-3 embryos	42	26	Table 1
	Control	Number of good-quality day-3 embryos	29	10	Table 1
	Melatonin 1 nmol/L (0-month cryopreservation)	Number of blastocysts	42	17	Table 1
	Control	Number of blastocysts	29	5	Table 1
	Melatonin 1 nmol/L (6-month cryopreservation)	Fertilization rate	57	37	Table 1
	Control	Fertilization rate	55	32	Table 1
	Melatonin 1 nmol/L (6-month cryopreservation)	Cleavage rate	37	25	Table 1
	Control	Cleavage rate	32	22	Table 1
	Melatonin 1 nmol/L (6-month cryopreservation)	Number of good-quality day-3 embryos	25	12	Table 1
	Control	Number of good-quality day-3 embryos	22	4	Table 1
	Melatonin 1 nmol/L (6-month cryopreservation)	Number of blastocysts	25	7	Table 1
	Control	Number of blastocysts	22	2	Table 1
Zhang et al., 2024	Melatonin 1 nmol/L (donor oocytes)	Oocyte survival rate	336	316	Table 2
	Control	Oocyte survival rate	636	569	Table 2
	Melatonin 1 nmol/L (donor oocytes)	Fertilization rate	316	263	Table 2
	Control	Fertilization rate	569	472	Table 2
	Melatonin 1 nmol/L (donor oocytes)	Cleavage rate	263	250	Table 2

Control	Cleavage rate	472	428	Table 2
Melatonin 1 nmol/L (donor oocytes)	Number of good-quality embryos	250	156	Table 2
Control	Number of good-quality embryos	428	222	Table 2
Melatonin 1 nmol/L (donor oocytes)	Number of blastocysts	250	113	Table 2
Control	Number of blastocysts	428	217	Table 2
Melatonin 1 nmol/L (donor oocytes)	Number of good-quality blastocysts	250	64	Table 2
Control	Number of good-quality blastocysts	428	95	Table 2
Melatonin 1 nmol/L (donor oocytes)	Biochemical pregnancy rate	37	21	Table 2
Control	Biochemical pregnancy rate	82	33	Table 2
Melatonin 1 nmol/L (donor oocytes)	Clinical pregnancy rate	37	21	Table 2
Control	Clinical pregnancy rate	82	30	Table 2
Melatonin 1 nmol/L (donor oocytes)	Live birth rate	37	13	Table 2
Control	Live birth rate	82	21	Table 2
Melatonin 1 nmol/L (donor oocytes)	Miscarriage rate	37	3	Table 2
Control	Miscarriage rate	82	8	Table 2
Melatonin 1 nmol/L (donor oocytes)	Implantation rate	47	23	Table 2
Control	Implantation rate	125	35	Table 2
Melatonin 1 nmol/L (autologous oocytes)	Oocyte survival rate	233	225	Table 2
Control	Oocyte survival rate	231	206	Table 2
Melatonin 1 nmol/L (autologous oocytes)	Fertilization rate	225	207	Table 2
Control	Fertilization rate	206	176	Table 2

	Melatonin 1 nmol/L (autologous oocytes)	Cleavage rate	207	189	Table 2
	Control	Cleavage rate	176	163	Table 2
	Melatonin 1 nmol/L (autologous oocytes)	Number of good-quality embryos	187	116	Table 2
	Control	Number of good-quality embryos	163	69	Table 2
	Melatonin 1 nmol/L (autologous oocytes)	Number of blastocysts	187	84	Table 2
	Control	Number of blastocysts	163	65	Table 2
	Melatonin 1 nmol/L (autologous oocytes)	Number of good-quality blastocysts	187	61	Table 2
	Control	Number of good-quality blastocysts	163	34	Table 2
	Melatonin 1 nmol/L (autologous oocytes)	Biochemical pregnancy rate	21	14	Table 2
	Control	Biochemical pregnancy rate	21	9	Table 2
	Melatonin 1 nmol/L (autologous oocytes)	Clinical pregnancy rate	21	13	Table 2
	Control	Clinical pregnancy rate	21	8	Table 2
	Melatonin 1 nmol/L (autologous oocytes)	Ongoing pregnancy rate	21	6	Table 2
	Control	Ongoing pregnancy rate	21	2	Table 2
	Melatonin 1 nmol/L (autologous oocytes)	Live birth rate	21	6	Table 2
	Control	Live birth rate	21	4	Table 2
	Melatonin 1 nmol/L (autologous oocytes)	Miscarriage rate	21	1	Table 2
	Control	Miscarriage rate	21	2	Table 2
Zhang et al., 2021	Melatonin 1 nmol/L	Oocyte survival rate	51	48	Table 1
	Control	Oocyte survival rate	55	48	Table 1
	Melatonin 1 nmol/L	Fertilization rate	48	43	Table 1

	Control	Fertilization rate	48	30	Table 1
	Melatonin 1 nmol/L	Cleavage rate	43	41	Table 1
	Control	Cleavage rate	30	27	Table 1
	Melatonin 1 nmol/L	Number of good-quality day-3 embryos	41	27	Table 1
	Control	Number of good-quality day-3 embryos	27	7	Table 1
	Melatonin 1 nmol/L	Number of blastocysts	41	16	Table 1
	Control	Number of blastocysts	27	3	Table 1
Zou et al., 2020	Melatonin 10^4 nmol/L	Number of mature oocytes	105	86	Table 2
	Control	Number of mature oocytes	88	67	Table 2
	Melatonin 10^4 nmol/L	Fertilization rate	86	68	Table 2
	Control	Fertilization rate	67	51	Table 2
	Melatonin 10^4 nmol/L	Cleavage rate	68	67	Table 2
	Control	Cleavage rate	51	49	Table 2
	Melatonin 10^4 nmol/L	Number of blastocysts	67	33	Table 2
	Control	Number of blastocysts	49	12	Table 2
	Melatonin 10^4 nmol/L	Number of good-quality blastocysts	67	19	Table 2
	Control	Number of good-quality blastocysts	49	1	Table 2
Yang et al., 2023	Melatonin 10^4 nmol/L	Number of mature oocytes	54	41	Table 2
	Control	Number of mature oocytes	55	24	Table 2
	Melatonin 10^4 nmol/L	Fertilization rate	41	31	Table 2
	Control	Fertilization rate	24	18	Table 2
	Melatonin 10^4 nmol/L	Cleavage rate	31	30	Table 2
	Control	Cleavage rate	18	15	Table 2
	Melatonin 10^4 nmol/L	Number of good-quality embryos	30	15	Table 2
	Control	Number of good-quality embryos	15	5	Table 2

	Melatonin 10^4 nmol/L	Number of blastocysts	30	16	Table 2
	Control	Number of blastocysts	15	2	Table 2
	Melatonin 10^4 nmol/L	Number of good-quality blastocysts	30	6	Table 2
	Control	Number of good-quality blastocysts	15	0	Table 2
Li et al., 2019	Melatonin 10^4 nmol/L	Number of mature oocytes	32	20	Table 1
	Control	Number of mature oocytes	37	17	Table 1
Hao et al., 2017	Melatonin 10^4 nmol/L	Number of mature oocytes	159	112	Table 3
	Control	Number of mature oocytes	135	101	Table 3
	Melatonin 10^4 nmol/L	Fertilization rate	112	101	Table 3
	Control	Fertilization rate	101	80	Table 3
	Melatonin 10^4 nmol/L	Cleavage rate	101	93	Table 3
	Control	Cleavage rate	80	73	Table 3
	Melatonin 10^4 nmol/L	Number of blastocysts	93	26	Table 3
	Control	Number of blastocysts	73	14	Table 3
	Melatonin 10^4 nmol/L	Number of good-quality blastocysts	93	14	Table 3
	Control	Number of good-quality blastocysts	73	2	Table 3
Zhu et al., 2022b	Melatonin 10^4 nmol/L	Fertilization rate	58	51	Table 1
	Control	Fertilization rate	61	52	Table 1
	Melatonin 10^4 nmol/L	Cleavage rate	51	45	Table 1
	Control	Cleavage rate	52	43	Table 1
	Melatonin 10^4 nmol/L	Number of blastocysts	45	5	Table 1
	Control	Number of blastocysts	43	7	Table 1
	Melatonin 10^4 nmol/L	Number of good-quality blastocysts	45	2	Table 1
	Control	Number of good-quality blastocysts	43	2	Table 1
Wei et al.,	Melatonin 10^4 nmol/L	Immature oocyte rate	115	85±6.16	Table 1

2013	Control	Immature oocyte rate	121	86.5±5.82	Table 1
Akter et al., 2023	Melatonin 3 mg/day	Number of mature follicles	40	1.9±0.92	Table 3
	Control	Number of mature follicles	34	1.02±0.9	Table 3
	Melatonin 3 mg/day	Dominant follicle diameter (mm)	40	19.43±1.72	Table 3
	Control	Dominant follicle diameter (mm)	34	16.96±5.27	Table 3
	Melatonin 3 mg/day	Ovulation rate (reported RR)	40	1.34 (0.09 - 1.68)	Table 4
	Control	Ovulation rate	34	/	Table 4
	Melatonin 3 mg/day	Clinical pregnancy rate (reported RR)	40	2.18 (0.37 - 3.51)	Table 4
	Control	Clinical pregnancy rate	34	/	Table 4
Jamilian et al., 2019	Melatonin 10 mg/day	Total antioxidant capacity (mmol/L) (reported β estimate)	28	106.07 (62.87 - 149.28)	Table 4
	Control	Total antioxidant capacity (mmol/L)	28	/	Table 4
Shabani et al., 2019	Melatonin 10 mg/day	Fasting blood glucose (mg/dL) (reported β estimate)	29	-0.44 (-2.38 - 1.5)	Table 3
	Control	Fasting blood glucose (mg/dL)	29	/	Table 3
	Melatonin 10 mg/day	Triglycerides (mg/dL) (reported β estimate)	29	-7.9 (17.54 - 1.72)	Table 3
	Control	Triglycerides (mg/dL)	29	/	Table 3
	Melatonin 10 mg/day	Total cholesterol (mg/dL) (reported β estimate)	29	-7.96 (-13.75 - -2.17)	Table 3
	Control	Total cholesterol (mg/dL)	29	/	Table 3
	Melatonin 10 mg/day	LDL cholesterol (mg/dL) (reported β estimate)	29	-5.88 (-11.42 - -0.33)	Table 3
	Control	LDL cholesterol (mg/dL)	29	/	Table 3
	Melatonin 10 mg/day	HDL cholesterol (mg/dL) (reported β estimate)	29	-0.39 (-2.46 - 1.67)	Table 3
	Control	HDL cholesterol (mg/dL)	29	/	Table 3
Alizadeh et al., 2021	Melatonin 6 mg/day	Fasting blood glucose (mg/dL) (change from baseline)	21	-1.05±9.05	Table 3

	Control	Fasting blood glucose (mg/dL) (change from baseline)	20	0.45±6.56	Table 3
	Melatonin 6 mg/day	Triglycerides (mg/dL) (change from baseline)	21	-3.01±55.45	Table 3
	Control	Triglycerides (mg/dL) (change from baseline)	20	-16.33±44.88	Table 3
	Melatonin 6 mg/day	Total cholesterol (mg/dL) (change from baseline)	21	-0.73±20.94	Table 3
	Control	Total cholesterol (mg/dL) (change from baseline)	20	-7.67±19.54	Table 3
	Melatonin 6 mg/day	LDL cholesterol (mg/dL) (change from baseline)	21	-2.7±17.26	Table 3
	Control	LDL cholesterol (mg/dL) (change from baseline)	20	-6.86±18.05	Table 3
	Melatonin 6 mg/day	HDL cholesterol (mg/dL) (change from baseline)	21	2.76±4.81	Table 3
	Control	HDL cholesterol (mg/dL) (change from baseline)	20	1.19±2.91	Table 3
	Melatonin 6 mg/day	Total antioxidant capacity (mmol/L) (change from baseline)	21	0.08±0.26	Table 3
	Control	Total antioxidant capacity (mmol/L) (change from baseline)	20	-0.13±0.2	Table 3
Lim et al., 2019	PCOS (3 phenotypes)	Morning serum melatonin level (pg/mL)	68	44.4±26	Table 3
	PCOS (2 phenotypes)	Morning serum melatonin level (pg/mL)	41	56±35.9	Table 3
	Control (0 phenotypes)	Morning serum melatonin level (pg/mL)	122	37.5±26.1	Table 3
Terzieva et al., 2013	PCOS	Morning serum melatonin level (pg/mL)	30	49.37±3.79	Table 2
	Control	Morning serum melatonin level (pg/mL)	25	42.91±9.38	Table 2
	PCOS	Nocturnal serum melatonin level (pg/mL)	30	88.63±7.22	Table 2
	Control	Nocturnal serum melatonin level (pg/mL)	25	103.32±8.51	Table 2
Abduljabbar et al., 2020	PCOS	Morning serum melatonin level (ng/mL)	60	61.21±13.78	Table 2
	Control	Morning serum melatonin level (ng/mL)	30	28.61±6.73	Table 2
Jain et al.,	PCOS	Nocturnal serum melatonin level (pg/mL)	50	63.27±10.97	Table 1

2013	Control	Nocturnal serum melatonin level (pg/mL)	50	32.51±10.55	Table 1
Li et al., 2022	PCOS	Follicular fluid melatonin level (pg/mL)	35	49.137±1.151	Table 1
	Control	Follicular fluid melatonin level (pg/mL)	36	54.455±1.303	Table 1
Luboshitzky et al., 2003	PCOS	Urinary 6-sulfatoxymelatonin (μg/24 h)	12	52.6±20.3	Table 2
	Control	Urinary 6-sulfatoxymelatonin (μg/24 h)	15	30.5±6.5	Table 2
Shreeve et al., 2013	PCOS	Nocturnal urinary 6-sulfatoxymelatonin (pg/mL)	15	60.3±30.6	Result section, p.1351
	Control	Nocturnal urinary 6-sulfatoxymelatonin (pg/mL)	18	37.7±21.5	Result section, p.1351
Sadeghpour et al., 2025	Melatonin 3 mg/day	Number of oocytes retrieved	35	7.38±1.65	Table 2
	Control	Number of oocytes retrieved	33	3.62±0.89	Table 2
	Melatonin 3 mg/day	Number of mature oocytes	35	5.91±1.68	Table 2
	Control	Number of mature oocytes	33	2.35±0.59	Table 2
	Melatonin 3 mg/day	Serum estradiol on hCG trigger day (pg/mL)	35	1867.35±807.41	Table 2
	Control	Serum estradiol on hCG trigger day (pg/mL)	33	1236.88±560.97	Table 2
	Melatonin 3 mg/day	Total HMG dose (IU)	35	3917.21±886.45	Table 2
	Control	Total HMG dose (IU)	33	3805.53±864.59	Table 2
	Melatonin 3 mg/day	Days of ovarian stimulation	35	10.17±0.97	Table 2
	Control	Days of ovarian stimulation	33	11.85±1.31	Table 2
	Melatonin 3 mg/day	Fertilization rate (%)	35	65.68±14.45	Table 2
	Control	Fertilization rate (%)	33	43.67±12.62	Table 2
	Melatonin 3 mg/day	Good-quality embryo rate (%)	35	43.54±16.21	Table 2
	Control	Good-quality embryo rate (%)	33	29.1±11.22	Table 2
Jahromi et al., 2017	Melatonin 3 mg/day	Number of mature oocytes	32	5.38±2.37	Table 2
	Control	Number of mature oocytes	34	3.7±2.7	Table 2
	Melatonin 3 mg/day	Serum estradiol on hCG trigger day (pg/mL)	32	2133±1272	Table 2

	Control	Serum estradiol on hCG trigger day (pg/mL)	34	1193±1396	Table 2
	Melatonin 3 mg/day	Total HMG dose (IU)	32	3975±1269	Table 2
	Control	Total HMG dose (IU)	34	3860±1384	Table 2
	Melatonin 3 mg/day	Days of ovarian stimulation	32	11±2.3	Table 2
	Control	Days of ovarian stimulation	34	10.3±2.4	Table 2
	Melatonin 3 mg/day	Clinical pregnancy rate	32	2	Table 2
	Control	Clinical pregnancy rate	34	1	Table 2
	Melatonin 3 mg/day	Miscarriage rate	32	2	Table 2
	Control	Miscarriage rate	34	1	Table 2
Li et al., 2016	Melatonin 1-3 mg/day	FSH (mIU/mL)	64	15.49±2.3	Table 2
	Control	FSH (mIU/mL)	64	47.49±2.3	Table 2
	Melatonin 1-3 mg/day	LH (mIU/mL)	64	10.4±2.1	Table 2
	Control	LH (mIU/mL)	64	27.9±0.42	Table 2
	Melatonin 1-3 mg/day	Estradiol (pg/mL)	64	142.7±33.4	Table 2
	Control	Estradiol (pg/mL)	64	76.4±21.8	Table 2
	Melatonin 1-3 mg/day	Reactive oxygen species (ng/mL)	64	58.5±22.7	Table 3
	Control	Reactive oxygen species (ng/mL)	64	211.2±13.3	Table 3
	Melatonin 1-3 mg/day	VEGF (ng/mL)	64	10.7±9.4	Table 3
	Control	VEGF (ng/mL)	64	56.5±12.1	Table 3

*Outcome data are presented as mean ± SD, mean (95% CI), median [P25, P75], n, percentage (%), RR (95% CI), or β (95% CI). Sample size represents the number of participants, oocytes, embryos, treatment cycles, or embryo-transfer cycles, as applicable to the corresponding outcome.

61. Mousavi, R.; Alizadeh, M.; Asghari Jafarabadi, M.; Heidari, L.; Nikbakht, R.; Babaahmadi Rezaei, H.; Karandish, M. Effects of Melatonin and/or Magnesium Supplementation on Biomarkers of Inflammation and Oxidative Stress in Women with Polycystic Ovary Syndrome: a Randomized, Double-Blind, Placebo-Controlled Trial. *Biological Trace Element Research* **2022**, 200, 1010-1019, doi:10.1007/s12011-021-02725-y.
62. Vakhlova, O.S.; Oboskalova, T.A.; Kvashnina, E.V.; Mukhlynina, E.A. CLINICAL EFFICACY OF IN VITRO FERTILIZATION IN PATIENTS TREATED WITH MELATONIN DURING PRECONCEPTION CARE. *Akusherstvo i Ginekologiya (Russian Federation) 2022*, **2022**, 67-74, doi:10.18565/aig.2022.6.67-74.
63. Li, Y.; Liu, H.; Wu, K.; Liu, H.; Huang, T.; Chen, Z.J.; Zhao, S.; Ma, J.; Zhao, H. Melatonin promotes human oocyte maturation and early embryo development by enhancing clathrin-mediated endocytosis. *J Pineal Res* **2019**, 67, e12601, doi:10.1111/jpi.12601.
64. Lim, A.J.R.; Indran, I.R.; Kramer, M.S.; Yong, E.L. Phenotypic spectrum of polycystic ovary syndrome and their relationship to the circadian biomarkers, melatonin and cortisol. *Endocrinol Diabetes Metab* **2019**, 2, e00047, doi:10.1002/edm2.47.
65. Terzieva, D.D.; Orbetzova, M.M.; Mitkov, M.D.; Mateva, N.G. Serum melatonin in women with polycystic ovary syndrome. *Folia medica* **2013**, 55, 10-15, doi:10.2478/folmed-2013-0012.
66. Jain, P.; Jain, M.; Haldar, C.; Singh, T.B.; Jain, S. Melatonin and its correlation with testosterone in polycystic ovarian syndrome. *J Hum Reprod Sci* **2013**, 6, 253-258, doi:10.4103/0974-1208.126295.
